# Integrated time-lapse and single-cell transcription studies highlight the variable and dynamic nature of human hematopoietic cell fate commitment

**DOI:** 10.1371/journal.pbio.2001867

**Published:** 2017-07-27

**Authors:** Alice Moussy, Jérémie Cosette, Romuald Parmentier, Cindy da Silva, Guillaume Corre, Angélique Richard, Olivier Gandrillon, Daniel Stockholm, András Páldi

**Affiliations:** 1 Ecole Pratique des Hautes Etudes, PSL Research University, UMRS 951, INSERM, Univ-Evry, Evry, France; 2 Genethon, Evry, France; 3 Laboratoire de Biologie et de Modélisation de la Cellule, Ecole Normale Supérieure de Lyon, CNRS, Université de Lyon, Lyon, France; Institute for Systems Biology, United States of America

## Abstract

Individual cells take lineage commitment decisions in a way that is not necessarily uniform. We address this issue by characterising transcriptional changes in cord blood-derived CD34+ cells at the single-cell level and integrating data with cell division history and morphological changes determined by time-lapse microscopy. We show that major transcriptional changes leading to a multilineage-primed gene expression state occur very rapidly during the first cell cycle. One of the 2 stable lineage-primed patterns emerges gradually in each cell with variable timing. Some cells reach a stable morphology and molecular phenotype by the end of the first cell cycle and transmit it clonally. Others fluctuate between the 2 phenotypes over several cell cycles. Our analysis highlights the dynamic nature and variable timing of cell fate commitment in hematopoietic cells, links the gene expression pattern to cell morphology, and identifies a new category of cells with fluctuating phenotypic characteristics, demonstrating the complexity of the fate decision process (which is different from a simple binary switch between 2 options, as it is usually envisioned).

## Author summary

Hematopoietic stem cells are classically defined as a specific category of cells at the top of the hierarchy that can differentiate all blood cell types following step-by-step the instructions of a deterministic program. We have analysed this process, and our findings support a much more dynamic view than previously described. We apply time-lapse microscopy coupled to single-cell molecular analyses in human hematopoietic stem cells and find that fate decision is not a unique, programmed event but a process of spontaneous variation and selective stabilisation reminiscent of trial–error processes. We show that each cell explores (at its own pace and independently of cell division) many different possibilities before reaching a stable combination of genes to be expressed. Our results suggest, therefore, that multipotency seems to be more like a transitory state than a feature of a specific cell category.

## Introduction

Hematopoietic stem and progenitor cells (HSPCs) give rise to all the cellular components of blood. The major stages of differentiation and the key genes participating in this process are now well characterised [1]. According to the classical view, haematopoiesis is a hierarchically organised process of successive fate commitments, in which differentiation potential is progressively restricted in an orderly way over cell divisions. There are several variants of the model [2–6]. In all cases, the first fate decision is a binary choice taken by multipotent progenitors (MPPs), which leads to 2 different committed progenitors (for the purpose of simplicity, these progenitors are designated here as common myeloid progenitors [CMP] and common lymphoid progenitors [CLP]). In molecular terms, the choice is believed to be the result of the strictly regulated activation of master regulator genes and their underlying transcriptional network [7]. However, the strict hierarchical logic of classical models has recently been challenged by a number of in vivo and in vitro studies [8–10]. Single-cell gene expression studies have revealed a much higher heterogeneity of cell subtypes than can be detected using a combination of surface markers [11]. It is not surprising that the number of identifiable cell types increases with the resolution of the detection method. Although correct cell type classification is a key step in understanding the cell fate decision issue, it cannot reveal the dynamic features of the fate commitment process and leaves a number of unanswered questions. Do different phenotypic forms represent different cell types or different stages of the same process? How does the transition between the forms occur? How long does it take?

Until recently, fully deterministic explanations were predominant, but recent studies have suggested other alternatives. Two different possibilities have been put forward. According to the first, the commitment process starts with the sporadic, independent activation of genes within the same cell. The simultaneous stochastic expression of regulatory genes specifying different lineages creates a multiprimed intermediate state that enables these cells to choose 1 of the lineages [12–16]. A coherent lineage-specific expression profile would then emerge from this metastable state. According to the second, commitment is preceded by transcriptome fluctuations between different lineage-biased states [17–19]. Surprisingly, the time scale of transformations related to the cellular fate decision process remains largely unexplored. The transcriptome of the same cell can be analysed only once, because the cell is destroyed by RNA extraction. Therefore, indirect approaches are required to identify trends and patterns in time series.

We addressed the issue of the dynamics and the time scale of the commitment process by integrating single-cell quantitative reverse transcription polymerase chain reaction (qRT-PCR), cell division history, and morphological changes determined by time-lapse analysis. Contrary to the common strategy consisting of isolating defined cell subpopulations with the help of specific surface markers and characterising their gene expression profiles at the single-cell level [20], we used an alternative approach. Individual cells were randomly isolated from the heterogeneous cord blood CD34+ cell fraction at different time points after cytokine stimulation, and their gene expression profiles were determined using single-cell qRT-PCR. The data provided a series of snapshots, showing the actual statistical distribution of single-cell gene expression patterns across the whole population. The structure of the population at the successive time points was revealed by unsupervised classification of the expression profiles according to their similarity using multiparametric approaches. The progression of the fate commitment process was deduced from the evolution of the population structure. At the same time, using time-lapse microscopy, we tracked randomly isolated individual CD34+ cells and their progeny for several days after cytokine stimulation. We recorded the division history and the morphological changes of each cell in the clones. The population structure was deduced on the basis of the statistical analysis of these observations. The efficiency of the time-lapse approach in investigating cell fate decisions has recently been shown [21]. To reinforce this approach, the time-lapse and gene expression data were integrated into a coherent scenario. This was done by using CD133 protein expression levels to isolate cells with 1 transcription profile or the other and directly record their dynamic phenotype, thereby providing a direct link between dynamic phenotype and transcription profile.

Altogether, our results revealed that fate decision is a dynamic, fluctuating process that is more complex than a simple binary switch between 2 options, as it is usually envisioned.

## Results

### Single-cell gene expression

**Fig 1:**
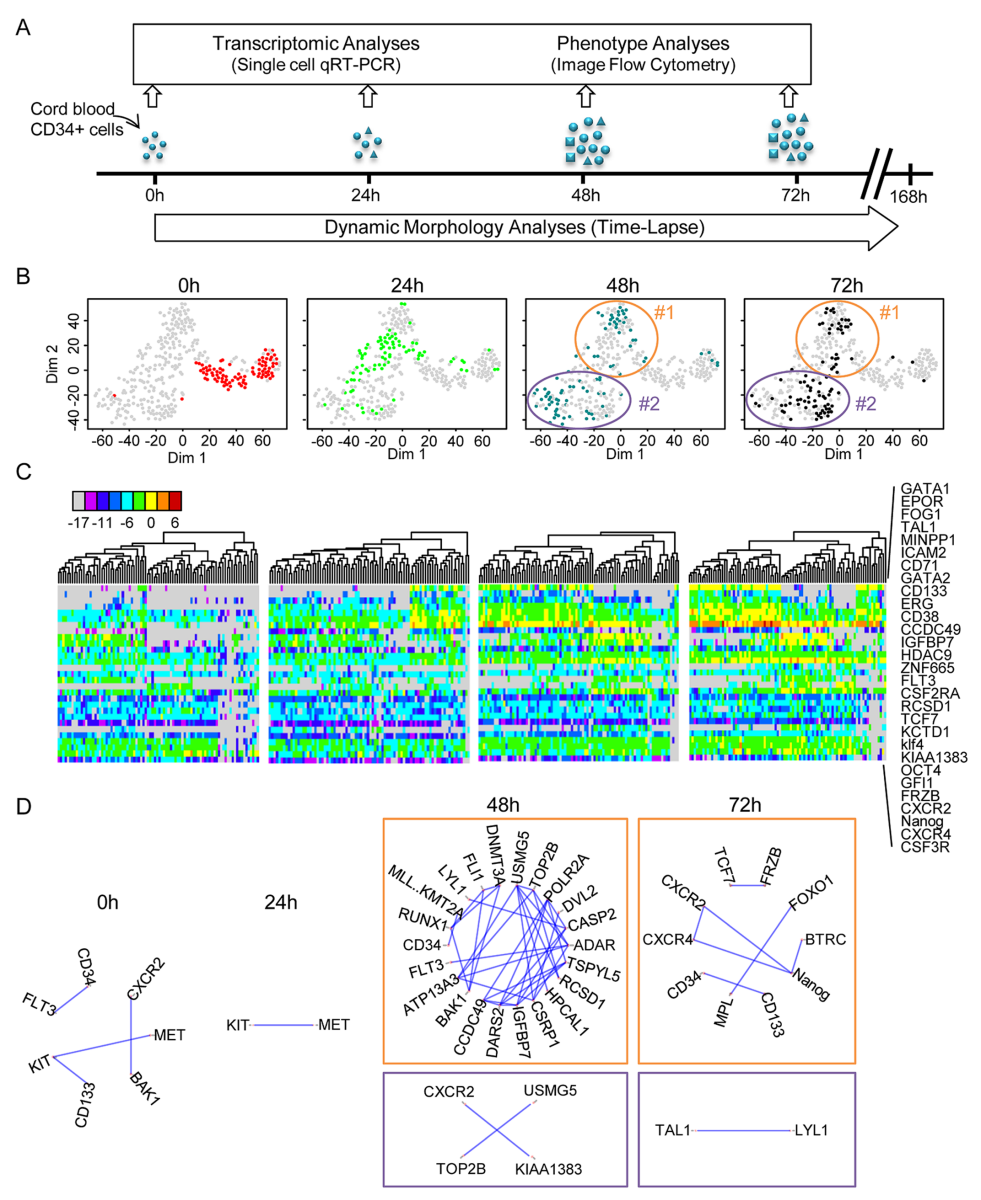
Transcriptional profile of cord blood-derived CD34+ cells at t = 0 h, t = 24 h, t = 48 h, and t = 72 h after the beginning of the experiment. (A) CD34+ cells were isolated from human cord blood and cultured in serum-free medium with early acting cytokines. Single-cell quantitative reverse transcription polymerase chain reaction (qRT-PCR) was used to analyse single-cell transcription at 0 h, 24 h, 48 h and 72 h. At the same time, individual clones were continuously monitored using time-lapse microscopy. (B) t-distributed stochastic neighbour embedding (t-SNE) map of single-cell transcription data. The 4 panels show analysis of the same data set, with each point representing a single cell highlighted in different colours depending on the given time point. The 2 clusters identified by gap statistics at t = 48 h and t = 72 h are surrounded by an ellipse and numbered #1 and #2 for multipotent and common myeloid progenitor (CMP)-like cells. Note the rapid evolution of the expression profiles. (Underlying data can be found in S1 Data.) (C) A heat map representation of the expression levels of a subset of genes that strongly contributed to the differentiation of the different groups (as detected by principal component analysis [PCA]; see S2 Fig) and cluster analysis of expression profiles at the different time points show the rapid evolution of gene expression. The list of the genes used (shown on the right) includes well-known genes acting during hematopoietic differentiation but also many randomly selected genes. The colour code for expression levels is indicated below. (Underlying data can be found in S1 Data.) (D) Pairwise correlations between the genes analysed using single-cell quantitative reverse transcription polymerase chain reaction (qRT-PCR). Only the gene pairs with a Pearson correlation coefficient higher than 0.8 are indicated for each time point. The 2 clusters identified at t = 48 h and t = 72 h are represented separately. Note the transient increase of the average correlation in cluster #2 at t = 48 h, indicating a state transition. (Underlying data can be found in S1 Data.)

The transcriptional profile of individual cord blood CD34+ cells was determined at 0, 24, 48, and 72 h after the beginning of cytokine stimulation (Fig 1A). Single-cell qRT-PCR was used to quantify the mRNA levels of 90 different genes. A set of 32 genes was selected for their known function in the early differentiation of hematopoietic cells and was expected to inform on the functional state of the cells (see S1 Table). A second set of 54 genes was chosen randomly from a list of genes known to be expressed in the hematopoietic lineage [22,23]. These genes provided an assessment of the overall transcriptional activity of the genome. Five additional genes were added to the list for their role in maintaining the pluripotent state in embryonic stem cells. A heat map of all data and a violin plot of the expression profile of each gene at the 4 separate time points are shown in S1 Fig and S2 Fig. The normalized single-cell quantitative gene expression data obtained for the different time points were merged into a single database and screened for subpopulations by k-means clustering. The number of statistically distinguishable groups was inferred using gap statistics [24]. The groups were visualised on heat maps and on a 2D plot using t-distributed stochastic neighbour embedding (t-SNE) [25]. Although every cell had a unique gene expression pattern, this approach enabled us to clearly identify subgroups of cells in the population on the basis of the statistical similarity of their gene expression patterns (Fig 1B).

Nonstimulated CD34+ cells isolated from cord blood represented the t = 0 h time point. A heat map of the single-cell transcriptional profiles of genes contributing significantly to the identification of subgroups (S1 Fig) showed that this population of cells was heterogeneous. Several genes reported to play a role in self-renewal, quiescence, and other stem cell functions (CD71, CD133, CXCR4, GATA2, and FLT3) were expressed sporadically and at variable levels in a fraction of cells. Genuine pluripotent stem cell genes were also expressed at low levels in a fraction of cells (NANOG, OCT4, KLF4). Nevertheless, no correlation was found between these genes (Fig 1D), and the statistical analysis did not reveal distinguishable expression patterns that could define cell types. The only detectable differences were donor-associated and probably reflected differences related to the processing of individual blood samples. Donor-specific differences disappeared at later stages.

The gene expression profile 24 h after the onset of cytokine stimulation was found to be fundamentally different to t = 0 h cells. Almost every cell responded to cytokine stimulation by increasing transcript levels and generating a unique gene expression pattern (Fig 1C). When represented on the 2D t-SNE (Fig 1B) and principal component analysis (PCA) plots (S3 Fig), the cells formed a single but dispersed cluster, well separated from the t = 0 h cells. In a fraction of cells, moderate to high transcription of previously nonexpressed hematopoietic regulator genes was observed in addition to that already seen at t = 0 h. For example, the expression of GATA1, GATA2, PU1, CD71, FOG1, CD133, or EPOR increased or was more frequent than at t = 0 h. In some cells, all these genes were expressed simultaneously. Nevertheless, no distinct subpopulations could be identified at the resolution of our approach. The pairwise correlation coefficients between genes remained low (Fig 1D). It is therefore likely that the patterns observed at 24 h resulted from essentially uncoordinated up-regulation of gene transcription and led to a highly heterogeneous cell population. This is a transition state reminiscent of the reported multilineage primed state with simultaneous expression of lineage-affiliated genes specifying alternative cell fates [12,15].

The first signs of coordinated differential gene expression appeared at t = 48 h after cytokine stimulation. At this stage, 2 distinct gene expression patterns emerged from the highly variable background of earlier stages. The 2 clusters are clearly distinguishable on the t-SNE plot (Fig 1B) and identified by gap statistics. They are also easily seen on the heat map representing gene expression levels (Fig 1C). Cluster #2 comprised cells with simultaneous expression of genes characteristic of CMPs such as GATA1 and EPOR [7]. The expression profile of the cells in cluster #1 was characterised by the strong expression of genes reported for multipotent cells (CD133, GFI1, KLF4, or FLT3) and the lack of expression of GATA1 and EPOR. Although this pattern is reminiscent of a self-renewing, multipotent profile, it is difficult to determine the exact identity of these cells at the level of resolution used in our study [26]. Typical genes for pluripotent stem cells like NANOG and OCT4 were expressed at moderate levels in many cells from both clusters (Fig 1C, S1 Fig and S2 Fig). Randomly selected genes were also good predictors for the 2 groups of cells. Only a small fraction of cells could not be classified in 1 of the 2 main clusters at t = 48 h (Fig 1B). The tendency observed at t = 48 h was further reinforced by t = 72 h. The cells in cluster #2 with CMP-like profiles represented more than half of all cells (Fig 1B and 1C). We observed a strong but transient increase in the number of highly correlated genes in this group (Fig 1D). Such an increase in the overall gene-to-gene correlation is a typical hallmark of imminent state transition in these cells [27–29]. Indirectly, this suggested that the cluster #1 profile was more in continuity with the previous profile observed at t = 24 h and that the cluster #2 profile at t = 48 h represented a transition to a new pattern.

Taken together, these single-cell gene expression observations revealed that the cell fate decision process in cytokine-stimulated CD34+ cord blood cells occurred during the first 2 d. Initially, each cell responds to cytokine stimulation with an uncoordinated change in gene expression, which is followed by the emergence of 2 distinct gene expression patterns reminiscent of the 2 known major types of hematopoietic progenitor cells. Although indications of this second change may appear as early as 24 h after stimulation, the 2 distinct gene expression patterns are clearly distinguishable at 48 h and consolidated by 72 h. By this stage, almost every cell seems to have adopted 1 profile or the other.

### Time-lapse tracking studies

**Fig 2:**
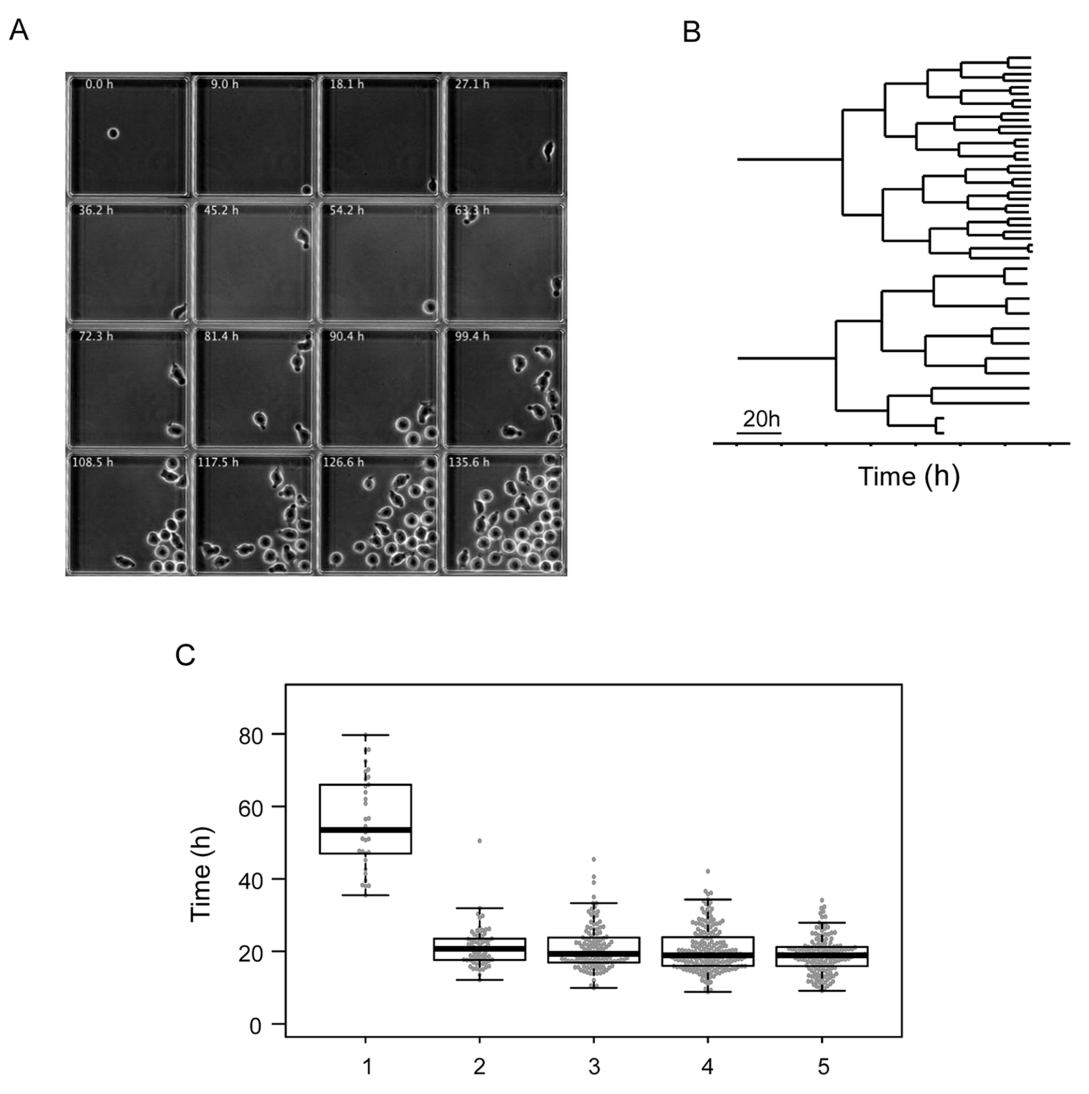
Time-lapse tracking of individual clones. (A) These frames, extracted from a representative time-lapse record, show a microwell containing a single founder cell, which divides to produce a clone. Each individual cell was tracked, and their morphological characteristics were recorded. (B) Two representative lineage pedigrees obtained from the time-lapse record. The strong difference in clone size observed at the end of the record is established gradually after the third cell division. (C) Box plot representation of cell cycle lengths obtained from the time-lapse records of every clone. Note the long first cell cycle. Subsequent cell cycles have comparable lengths, with a slight tendency to become shorter. (Underlying data can be found in S2 Data.)

In order to integrate the gene expression snapshots into a dynamic scenario, we made time-lapse records of individual CD34+ cells under in vitro conditions identical to those in the single-cell gene expression studies. We imaged individual cells in microwells at a rate of 60 frames per hour for 7 d (Fig 2A). Using a semiautomatic image analysis approach, we established individual clonal pedigrees and recorded cell cycle durations and major morphological changes. As shown in Fig 2B and 2C, the pedigrees of individual clones were highly variable but shared some general features. Some clones produced only a few cells during the observation period, while others proliferated faster and produced up to 30–40 siblings. We focused our attention on the first 3 generations. As reported for cells cultured in early acting cytokines [30], the first cell cycle was exceptionally long in all clones. The division of the founder cell occurred between 35 h and 80 h after the start of culturing, with the median cell cycle length being 58 h (Fig 2). We questioned whether the culturing of isolated cells in microwells, in which direct contact with the other cells was not possible, influenced cell cycle length. To measure the division rate in a population context, the cells cultured together were labelled using CellTrace Violet (CTV). The results (S4 Fig) showed that the cells had similar division profiles regardless of whether they were cultured individually or in population. The unusually long first cell cycle was particularly important when interpreting results. It implied that the transition from the initial to the multilineage primed transcription profile followed by 1 of the 2 types of progenitor-like profiles observed at 24 h and 48 h after CD34+ cell stimulation occurred during the life of the founder cell, before the first mitosis.

Previous studies have demonstrated that there is a connection between cell morphology and the differentiation potential of CD34+ cells. Two major morphological forms have been described in the CD34+ cord blood cell fraction. Polarised cells are capable of active motion with the help of lamellipodia and possess, on their opposite end, large protrusions called uropods. These cells have been found to retain primitive self-renewing and stem cell functions [31,32]. The second morphological type is round. These cells have been considered as already engaged in differentiation [31,32].

Time-lapse records revealed that the 2 cell morphologies were not permanent; most cells were able to switch between forms several times during the cell cycle. After recovering from the stress of isolation and manipulation, founder cells acquired polarised morphologies within a few hours, developing uropods and starting to move actively (see S1–S3 Movies). During the first cell cycle, cells mostly conserved the polarised form, and switches between the 2 morphologies were rare. As indicated above, the first cell division occurred (on average) at 58 h, and the average lengths of subsequent cell cycles were around 20 h to 22 h. The daughter (second generation) and granddaughter (third generation) cells were able to switch between the 2 morphologies at a much higher frequency compared to the founder cells. In order to quantify these events, we manually tracked each cell and recorded each switch. Representative profiles are presented in S5 Fig.

**Fig 3:**
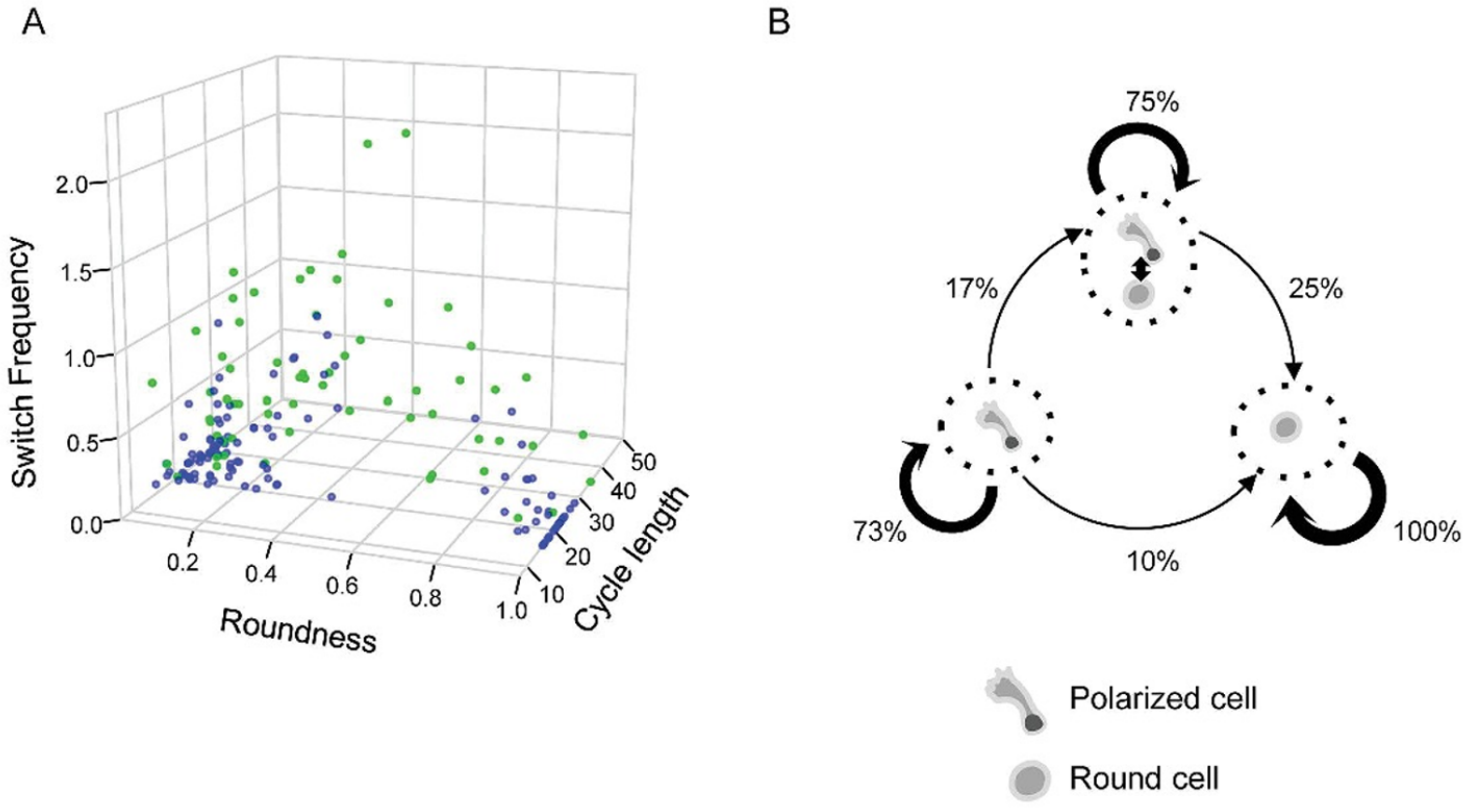
Quantitative analysis of dynamic phenotypes as determined by time-lapse data. (A) Association between the morphology, switch frequency, cell cycle length, and the type of cell divisions of second- and third-generation cells. Each point represents a single cell. Siblings with different dynamic behaviour and morphology (in green) are usually characterised by high switch frequencies. Siblings with similar dynamic behaviour and morphologies are shown in blue. The morphology is given as a ratio of time spent in round/polarised shape by a cell during the cell cycle. Switch frequency is given in number of morphological transformations per hour. Cell cycle length is in hours. (B) Dynamic phenotype change during the first 2 cell divisions as determined on the basis of time-lapse records. Three different dynamic phenotypes were identified: stable polarised, frequent switchers, and stable round. Cells tended to transmit dynamic phenotypes to daughter cells during cell division. Polarised and frequent switchers produced round cells, and frequent switchers were always produced by polarised mothers. Phenotypic change is not associated with asymmetric division; it can occur at any time in the cell cycle. Since round cells always produce round daughters, the whole process is biased and the proportion of this phenotype increases. (Underlying data can be found in S2 Data.)

In order to compare quantitatively the dynamic phenotype of cells, we calculated 3 parameters based on their dynamic profiles. The first parameter was calculated as a ratio of the time a given cell spent in a round shape compared to the time spent in a polarised shape. This parameter was close to 0 for stable polarised cells and 1 for stable round cells. Intermediate values correspond to the fraction of time cells spent in round shape. The second parameter was the frequency of morphological switches during the cell cycle. This parameter expressed the cell’s ability to maintain a stable morphology. The third parameter was the cell cycle length. When cells were represented as individual points in the space determined by the 3 parameters, we identified 3 major categories (Fig 3A). The first category included cells with mainly polarised shapes; the second category was composed of cells with predominantly round shapes; the cells in the third category switched shape frequently, generally fluctuating between both morphologies (Fig 3A).

When sister cell pairs were examined, it became obvious that many displayed very similar dynamic phenotypes. In some cases, periods of stable morphology and switching events coincided almost perfectly (S5 Fig). In other cases, the 2 sister cells behaved differently. In the most extreme cases, 1 sister cell adopted a stable round form and the other a stable polarised form immediately after division.

We calculated the frequency with which a cell with a given dynamic phenotype was produced by a mother cell with similar or dissimilar phenotype (Fig 3B). Maternal cells clearly tended to transmit the dynamic phenotype to daughter cells. We also observed the regularity with which phenotype changes occurred in daughter cells. Polarised cells were systematically produced by polarised cells. At lower probabilities, both polarised and fluctuating cells could produce stable round phenotype cells. Round cells always gave rise to round siblings (Fig 3B). Following these simple rules, the cumulative outcome of the process was the gradual increase of round cells in the population. Cells with fluctuating morphologies appeared to be an intermediate form between polarised and round cells. Since 25% of daughter cells conserved this phenotype, the fluctuating intermediate cells persisted in the population. On static snapshots, however, this category remained undetectable: only polarised and round cells were observed. A polarised form was considered to be a feature of multipotent cells and the round form a committed myeloid progenitor phenotype [31,32].

### Coupling the molecular and cellular scales

The dynamically fluctuating behaviour we have described here for the first time represents a transition between the 2 states and reflects a ‘hesitant’, incomplete fate-determination process. Since we detected only 2 major transcription profiles but observed 3 different dynamic behaviours, it is possible that ‘hesitant’ cells are not characterised by a clearly distinct transcription pattern. Morphology fluctuations may be accompanied by fluctuations in the transcript or protein levels of at least some key genes.

To test this assumption, we took advantage of the observation that the gene coding for the CD133 cell surface protein was expressed preferentially in 1 of the 2 transcription patterns detected at 48 h (Fig 1B and 1C). Previous reports have established that CD133 protein is typically present in cells with polarised forms and accumulates in the uropod [31–33]. We confirmed this using image cytometry and immunohistochemistry on fixed cells (S6 Fig). Cells expressing high levels of CD133 were mostly polarised, while those with low levels of CD133 were round (S6 Fig). This observation explicitly established a direct link between the cell morphology and the transcription patterns detected by single-cell qRT-PCR.

**Fig 4:**
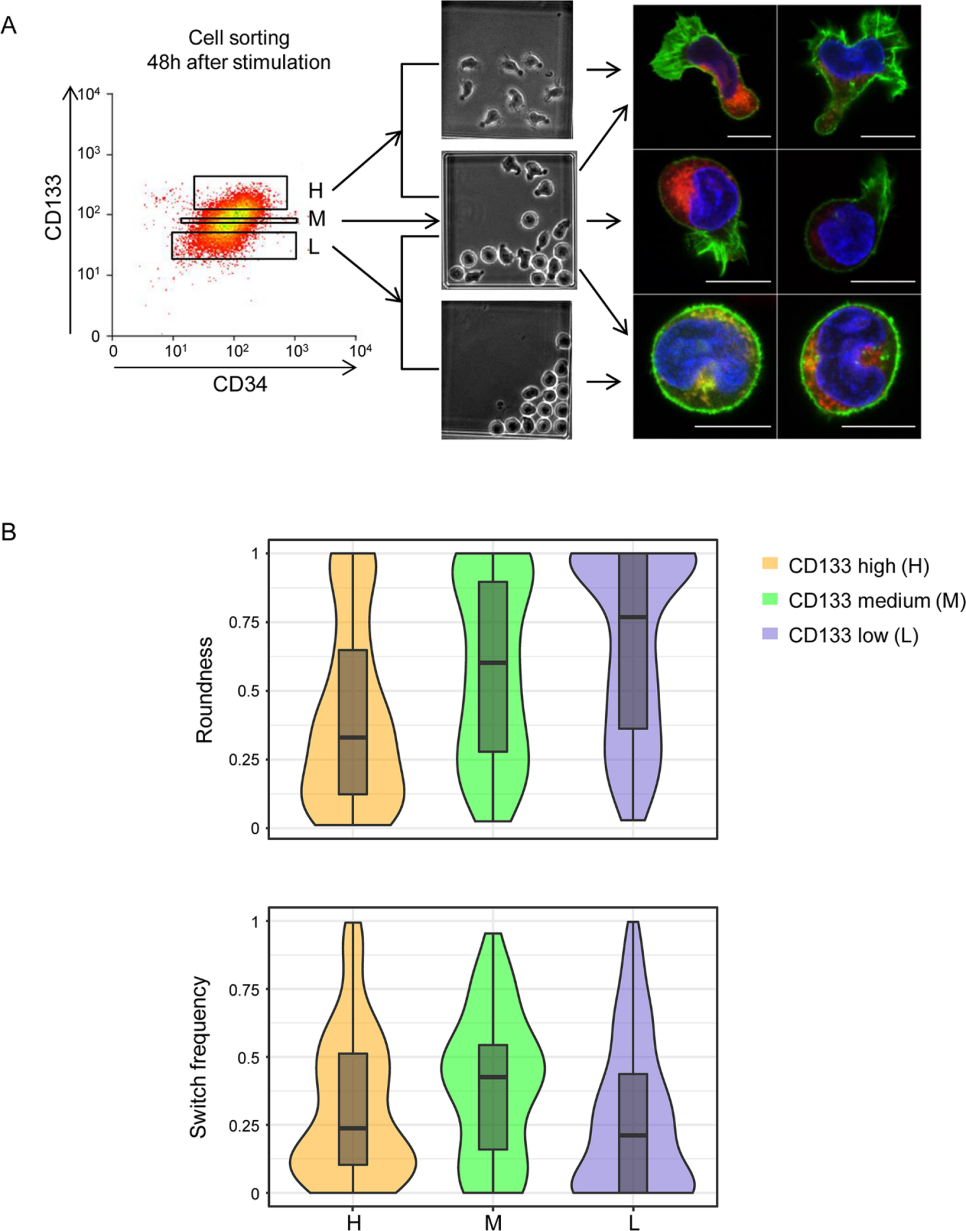
Isolation and time-lapse analysis of ‘high’, ‘medium’, and ‘low’ CD133-expressing expressing cells. (A) Cell-sorting strategy to isolate cells on the basis of the CD133 surface protein level. The sorted cells were cultured individually and tracked by time-lapse microscopy. They produced cells with a polarised, round, or fluctuating dynamic phenotype (illustrated by the middle panel). Examples of cells with different morphologies are shown on the right, as detected by confocal microscopy. Red: CD133 protein. Green: actin filaments detected by phalloidine. Blue: DNA. Note the preferential localisation of the CD133 protein in the uropods of polarised cells. Actin is concentrated in lammelipodia or evenly distributed in the periphery of round cells. (B) Quantitative evaluation of cell morphology and switch frequency. Distribution of the ‘roundness’ parameter (upper panel) indicates a gradual increase of the proportion of round cells between the ‘high’ and ‘low’ fraction. Distribution of the switch frequency as switch/h of sorted ‘high’, ‘medium’, and ‘low’ CD133 cells is shown in the lower panel. Note that the switch frequency is the highest in ‘medium’ CD133 cells. (Underlying data can be found in S2 Data.)

We therefore used the CD133 protein as a proxy for the isolation of a cell fraction enriched in either polarised or round cells and recorded their dynamic phenotype. The ‘high’, ‘medium’, and ‘low/negative’ CD133-expressing cell fractions were isolated 48 h after cytokine stimulation, put in culture, and tracked using time-lapse microscopy for an additional 48 h (Fig 4A). The fraction of the time the cell spent in round or in polarised shape, the switch frequency, and the division asymmetry of the tracked cells were quantified. The ‘high’ CD133-expressing cells and their progeny reproduced the 3 types of cells observed previously but in different proportions (Fig 4B). Most of the cells displayed stable polarised morphologies or were frequent switchers; only a few cells displayed stable round morphologies (Fig 4B). By contrast, the ‘low/negative’ cells produced either stable round progeny or cells with fluctuating morphologies (Fig 4B). The ‘medium’ CD133-expressing cells had a higher switch frequency, and both shapes were represented in a more equilibrated manner (Fig 4B).

These observations confirmed the idea that cells with stable round shapes were derived from cells with polarised shapes and high CD133 levels following a period in which they had a fluctuating phenotype. The fluctuations occurred in a bi-stable manner; the cells switched from 1 morphology to another and back rapidly without stable intermediate states (see S1–S3 Movies). The process of transformation did not correlate with the cell cycle; some cells reached a stable morphology rapidly, while others fluctuated over several cycles. The process was accompanied by a gradual decrease in CD133 protein levels in cells. We found no evidence that asymmetric divisions played a direct role in this process.

**Fig 5:**
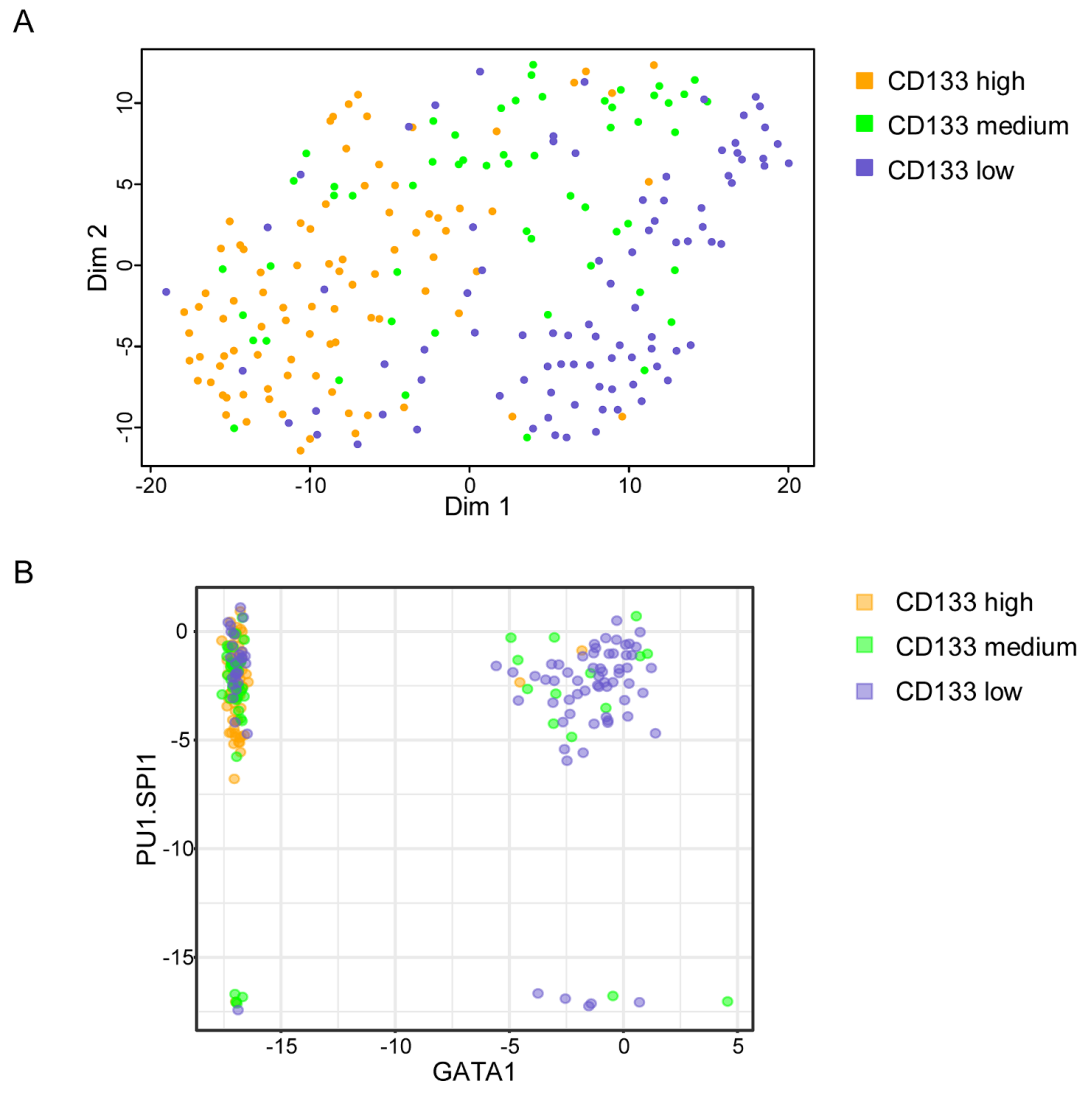
Single-cell gene expression in ‘high’, ‘medium’, and ‘low’ CD133 cells. (A) t-stochastic neighbour embedding (t-SNE) map of single-cell transcriptional data. Each point represents a single cell highlighted in a different colour for ‘high’, ‘medium’, and ‘low’ CD133 cells. ‘High’ and ‘low’ cells are in separated clusters corresponding to cluster #1 and #2 in Fig 1B. ‘Medium’ CD133 cells are distributed in and between these 2 clusters, indicating their intermediate character. (B) Scatter plot representation of PU1 and GATA1 expression in individual cells of the ‘high’, ‘medium’, and ‘low’ CD133 fraction. Note that GATA1 is not expressed in ‘high’ cells. Coexpression of the 2 genes is observed only in some ‘medium’ and ‘low’ cells. (Underlying data can be found in S1 Data.)

Next, we isolated individual cells with high, medium, and low/negative CD133 protein levels using a cell sorter and performed single-cell qRT-PCR analysis using the same gene panel used previously on the unsorted population. Fig 5A shows the t-SNE representation of the single-cell gene expression profiles. The heat map representation of the full set of gene expression results is shown in S7 Fig together with the PCA analysis. The ‘high’ and ‘low’ CD133 cells displayed different transcription profiles similar to clusters #1 and #2, respectively, found in cells of the unsorted population at t = 48 h (Fig 1). The cell fraction isolated on the basis of intermediate CD133 levels contained a large number of cells with intermediate transcription profiles, again supporting their dynamic transitory phenotype. When expression pattern of individual genes is examined (for example, CXCR4, CXCR2, DVL2, FOXO3, NANOG, ZNF665, or TSPYL5 [S8 Fig]), some of them displayed a very different distribution in the ‘medium CD133’ compared to the ‘high’ and ‘low’ CD133 cells. This further demonstrates that the ‘medium’ CD133 cells are more than simple intermediates between the ‘high’ and ‘low’ cells; they have their own dynamic transcription profile. One can conjecture that if the cell shape correlates with the CD133 level that correlates with the transcription profile, then those cells that change shape must also change transcription profile.

PU1 and GATA1 are well-known transcription factor-coding genes that play an important role in the specification of granulocytic–monocytic and erythroid–megakaryocitic cells [34]. It has been proposed that PU1 and GATA1 can cross-inhibit each other’s activity and generate a bi-stable switch between the 2 lineages [35], but more recent observations challenged this model [21]. Our analysis showed that ‘high’ CD133 cells express only PU1; ‘medium’ CD133 cells express PU1 only or coexpress the 2 genes; and ‘low’ CD133 cells express PU1 only, coexpress the 2 genes, or express GATA1 only (Fig 5B). This observation places the ‘medium’ CD133 cells as a possible intermediate between the cells expressing only PU1 and cells committed to different pathways, without providing evidence either for or against a direct competition between them.

### Single-cell transcription profile of the multipotent stage

**Fig 6:**
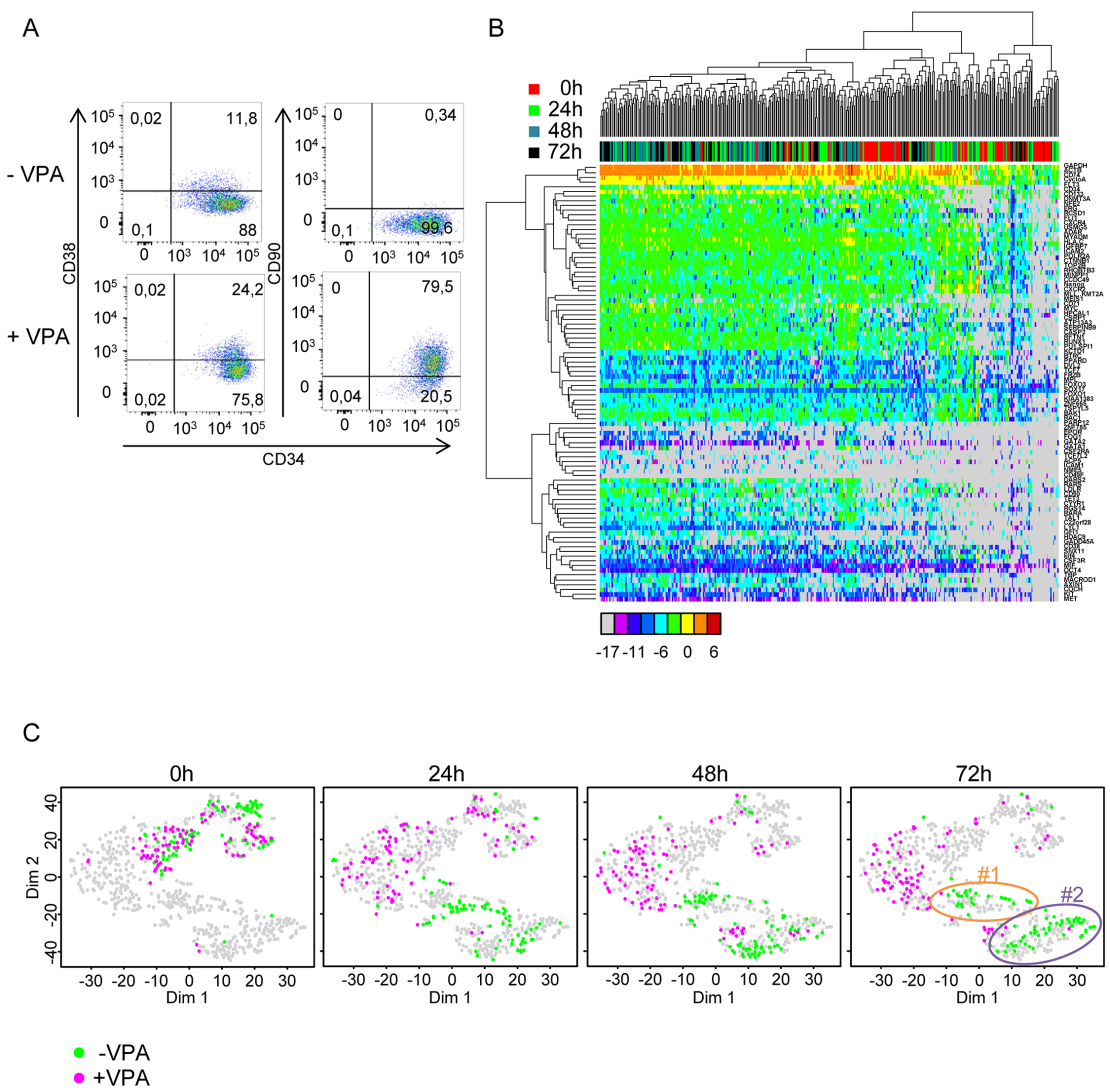
Transcriptional profile of cord blood-derived CD34+ cells treated with valproic acid (VPA) at t = 0 h, t = 24 h, t = 48 h, and t = 72 h after the beginning of the experiment as compared to untreated, normal control cells. (A) A cytometric analysis of the effect of VPA on cord blood CD34+ cells shows an increase in the CD90 protein in most cells, while the CD34 and CD38 markers remain essentially unchanged. (B) Heat map representation of the expression levels of 90 genes as determined by single-cell quantitative reverse transcription polymerase chain reaction (qRT-PCR) in VPA-treated cells at t = 0 h, t = 24 h, t = 48 h, and t = 72 h. The colour codes for the time points of cells are indicated on the right; the colour codes for expression levels are indicated below the heat map. Note the high heterogeneity and lack of clear clustering of the expression patterns. (C) t-distributed stochastic neighbour embedding (t-SNE) plot representation of transcription data obtained for VPA-treated cells compared to untreated normal cells (data for these cells are the same as in Fig 1). The gene expression data obtained in the 2 experiments were mapped together. Each point represents a single cell, and the cells at t = 0 h, t = 24 h, t = 48 h, and t = 72 h are highlighted separately in the 4 panels. The colour codes for VPA-treated (+VPA) and VPA-untreated (−VPA) are indicated below the panels. Clusters #1 and #2, identified at t = 48 h and t = 72 h in −VPA cells (see Fig 1), are indicated on the t = 72 h panel. Note the clear separation of the +VPA and −VPA cells at every time point except t = 24 h. Note also that +VPA cells do not contribute to clusters #1 and #2, indicating that they do not acquire expression profiles typical of these cells. (Underlying data can be found in S1 Data.)

In order to determine which of the observed phenotypes correspond to the multipotent stage, we took advantage of recent observations demonstrating that the inhibition of histone deacetylase (HDAC) activity with a pharmacological agent resulted in a substantial increase in their incidence in the CD34+ cord blood population [13,36,37]. We anticipated that this would increase the proportion of cells with transcription profiles typical of the multipotential phenotype. Since valproic acid (VPA) was shown to be the most efficient [36], we used this agent to treat CD34+ cord blood cells stimulated by cytokines as above, before sampling transcription profiles. The increase of the CD90 marker (as analysed by flow cytometry) confirmed that the VPA effect was already visible after 24 h and gradually grew stronger during subsequent steps (Fig 6A and S9 Fig). The expression of CD34 and CD38 markers remained unchanged (S9 Fig). Although we did not analyse the in vivo potential of these cells, based on previous reports, we considered them enriched for bona fide multipotent cells. We performed single-cell qRT-PCR at 0 h, 24 h, 48 h, and 72 h after the start of the experiment, as in control cells. At all 4 time points, cell populations were very heterogeneous. At each time point, the cells displayed a unique transcription profile (Fig 6B), and no identifiable transcription patterns appeared during the 72 h of the experiment, despite slight profile evolutions. Overall, transcription patterns in individual cells were reminiscent of the uncoordinated multilineage primed profile detected in control cells at 24 h, but the 2 groups clustered separately on t-SNE maps (Fig 6C). Since the cells did not divide during the first 48 h, the increase observed in the multipotent cell fraction could not result from the selective proliferation of an initially small subpopulation of cells. Instead, this occurred because cells already present in the population changed the expression of many genes in response to the VPA.

## Discussion

In this study, we aimed to identify the initial stages of fate commitment in the CD34+ cell fraction of human cord blood and determine the typical time scale for these events. Without cytokine stimulation, CD34+ cells remain quiescent and die after a few days in culture. Early acting cytokines allow these cells to survive, become metabolically active, and enter the cell cycle [38] without showing overt signs of differentiation during the first few days. This creates ideal conditions for studying early events. Our experimental design combined continuous time-lapse observations with snapshots of high-resolution single-cell transcriptome analysis. The data can be integrated in a dynamic fate-decision process scenario. Fate decision is necessarily accompanied by a change in the gene expression pattern. This is a multistep process. First, upon stimulation, cells rapidly reach the multiprimed state, which is characterised by a promiscuous gene expression pattern and predominantly polarised morphologies. This is an unstable phase, and 2 distinct transcription profiles start to emerge before the end of the first cell cycle. The process by which cells relax from a multiprimed to a more stable state is continuous and of variable length. Some cells reach stable morphology and a coherent, lineage-affiliated transcription profile by the end of the first cell cycle, which they then transmit to daughter cells. Other cells divide into unstable daughter cells with dynamic, ‘hesitant’ behaviour. This behaviour is characterised by fluctuations between polarised, actively moving amoeboid and round morphologies over several cell cycles, suggesting that instability can be transmitted mitotically. Although we have no formal evidence that the transcriptome of these cells also fluctuates, 2 observations suggest that this could be the case. First, we only found 2 established transcription profiles that correspond to polarised and round morphologies with ‘high’ and ‘low’ CD133 protein levels (Fig 1B). However, we observe 3 dynamic phenotypes, 1 of which is fluctuating. Second, cells isolated on the basis of having ‘medium’ CD133 protein levels represent a transition between the stable polarised and round morphology. We propose a dynamic scenario in which the initial stochastic multilineage primed state is followed by a period of relaxation and uncertain ‘hesitant’ phase of variable length with fluctuating transcriptomes [15,17] before a stable lineage committed state is reached. In addition, this scenario is also in accordance with the recent proposal that there exists a fraction of low-primed, undifferentiated cells called ‘CLOUD’-HSPCs in which that can fluctuate without passing through fixed, discrete states [10].

Increased stochastic variation in gene expression may be responsible for the rapid shift away from the initial quiescent state and lead to the uncommitted multilineage primed state [10,12,15,16]. Cell division is not required for this process; it occurs during the first cell cycle following stimulation. Cells on the path toward the new phenotype represent the committed state. The critical moment in this process is the transition between the 2 phenotypes, when the old gene network has broken down but the new network is not yet assembled. We consider that cells with fluctuating morphologies represent this transition state. The rapidity of the transition may be dependent on the time required for the new gene expression network to settle into a stable state. Since phenotypic stability of a cell lineage largely depends on the frequency of transcription initiation and the stability of the resulting mRNAs and proteins [39,40], the observed ‘hesitant’ phenotype might be the consequence of stochastic fluctuations due to rapid mRNA and protein turnover. The consolidation of the chromatin structure appears to be an essential element in this process, because, as shown in single-cell transcription studies, the HDAC inhibitor VPA delays the transition and blocks cells in a promiscuous gene expression pattern typical of a multilineage primed state. Indeed, HDAC inhibitors have been shown to increase gene expression stochasticity by increasing chromatin acetylation [41].

In summary, in this study, we identified the earliest phases of fate commitment in human cord blood CD34+ cells and assigned a time scale to this process. We demonstrated that the rapid initiation of the process occurs within a single cell cycle and is followed by a dynamic transition state of variable length that may span several cell cycles. Since experimental conditions were constant, the changes observed are likely to reflect cell-intrinsic processes, whereas the convergence toward a similar endpoint may reflect the constraints imposed by these conditions. From this perspective, fate decision appears to be a process of spontaneous variation/selective stabilisation reminiscent of trial–error learning, in which each cell explores many different possibilities at its own pace by expressing a large variety of genes before finding a stable combination corresponding to the actual environment. This is in remarkable agreement with earlier theoretical predictions and experimental work [42–46]. At least 3 independent theoretical models predicted the existence of an initial fluctuating phase during differentiation. According to the first theory, cell differentiation is a variation/selection process analogous to evolution [42,43]. Variations are created by stochastic fluctuations of gene expression, and some patterns are selectively stabilised through interactions with the environment and neighbouring cells. Another approach envisions cell phenotype as an attractor state in the parameter space defined by the gene expression network [47]. Differentiation is seen as a transition from 1 attractor to another and governed by the stochastic dynamics and self-organisation of the gene network. Finally, a dynamic system view of differentiation was independently proposed by Kaneko [48]. A common theme of these approaches is the prediction that differentiating cells must necessarily go through a dynamically fluctuating phase with oscillating gene expression. Several recent studies have reported on the existence of gene expression fluctuations during the critical state transitory phase of the differentiation process [15,29,46,49–51]. Our study goes a step further by demonstrating that the cellular phenotype also fluctuates during the critical transitory phase.

## Materials and methods

### Ethics statement

Human umbilical cord blood (UCB) was collected from placentas and/or umbilical cords obtained from Etablissement Français du Sang (EFS), Saint Louis Hospital, France or from Centre Hospitalier Sud Francilien, Evry, France in accordance with international ethical principles and French national law (bioethics law n° 2011–814) under declaration N° DC-201-1655 to the French Ministry of Research and Higher Studies.

### Human sample and cell culture

Human CD34+ cells were isolated from the mononuclear fraction of UCB samples using the autoMACSpro (Miltenyi Biotec, Paris, France) immunomagnetic cell separation system. They were then cryopreserved in Cryostor (StemCell, Paris, France) and stored in liquid nitrogen or used directly without freezing.

Cells were cultured at 37°C in a humidified atmosphere containing 5% CO_2_ in a 24-well plate in X-VIVO (Lonza) supplemented with 100 U/ml penicillin, 100 μg/ml streptomycin (Gibco, Thermo Scientific), 50 ng/ml h-FLT3, 25 ng/ml h-SCF, 25 ng/ml h-TPO, and 10 ng/ml h-IL3 (Miltenyi Biotec, Paris, France) final concentration. VPA (Sigma Aldrich) was used at a final concentration of 1.25 mM.

### Single-cell qRT-PCR

Single-cell qRT-PCR was carried out using the BioMark HD System (Fluidigm). Deltagenes assays (Life Technologies) were used at a final concentration of 500 nM for each of the 96 assays. Individual cells were sorted directly into a reverse transcription RT mix solution and spikes (Life Technologies) in a 96-well plate. RNA was denatured and reverse-transcribed. Twenty cycles of preamplification of 96 specific cDNA were performed by denaturing the cDNA at 96°C for 5 seconds, followed by annealing and extension at 60°C for 4 min. Unincorporated primers were cleaned up by Exonuclease I, and the preamplified products were diluted 5-fold. Amplification was performed with Evagreen supermix with low ROX (Bio-Rad) and inventoried DeltaGenes assays in 96.96 Dynamic Arrays on a BioMark HD System (Fluidigm). Cycle threshold (Ct) values were calculated from the system’s software (BioMark Real-Time PCR Analysis, Fluidigm).

### Single-cell data normalisation

Ct values obtained from the BioMark HD System (Fluidigm) were normalised with the help of 2 externally added controls (spike 1 and spike 4, Life Technologies) according to a set of rules provided below. For each gene, inconsistent readings or ‘Failed’ quality control readings were removed. Cells with failed or inconsistent detection of spikes were removed. Expression values were calculated by subtracting the gene Ct value from the geometric average of Ct values from spike 1 and spike 4 in the corresponding cell. An arbitrary differential cycle threshold (dCt) value of −17 was assigned for all the genes with a dCt value less than −17.

### Single-cell qRT-PCR data analysis

Analyses of qRT-PCR single-cell data were done with R software (R Core Team [2016]. R: A language and environment for statistical computing. R Foundation for Statistical Computing, Vienna, Austria, http://www.R-project.org/) using Heatmap3 [52], factomineR [53], k-means, and ggplot2 packages [54]. Correlation calculations were performed using custom R scripts. t-SNE and gap statistics calculations were performed as described by Grun et al. [55]

### Confocal microscopy

Images were obtained with a spectral confocal Leica SP8 scanning microscope (Leica Microsystems, Germany). 5.10^4^ cells were cultured in a 48-well plate in 200 μL prestimulation medium. After 72 h, 100 μL of 3% glutaraldehyde was added to the cell-containing well (1% final) for 15 min. Cells were washed twice with PBS 1X and incubated 2 h with 2 mg/mL NaBH_4_ at room temperature. Fcɤ receptors were saturated with Gamma Immune (Sigma Aldrich) for 5 min at 4°C (1:2 dilution). The cells were permeabilised with the fix/perm kit (BD-Biosciences), labeled for 20 min at 4°C with a 1:10 dilution of the mouse anti-human CD133-APC antibody (clone Ac133, Miltenyi Biotec), a 1:1,000 dilution of phalloidin–Tetramethylrhodamine B isothiocyanate (Sigma Aldrich) and stained with DAPI.

The images were acquired using a 63X PL APO CS2 1.40 NA oil immersion objective (Leica Microsystems, Germany). DAPI was excited with a 405-nm laser, TRITC with a 552-nm laser, and APC with a 635-nm laser. Finally, images were processed with a contrast enhancement algorithm (histogram equalisation) and a home-designed background subtraction algorithm.

### Microgrid cell culture

A polydimethylsiloxane (PDMS) microgrid array (Microsurfaces, Australia) of 1,024 microwells (125-μm width, 60-μm depth) was placed in a specialised culture dish divided into 4 parts (Hi-Q4, Ibidi, Germany). Each part of the dish was filled with cell culture medium. A suspension of 5 × 10^3^ cells per case was added at a concentration likely to lead to a high number of wells with a single cell.

### Time-lapse microscopy

The time-lapse microscopy protocol was previously described [56]. Time-lapse acquisitions were performed with the Biostation IM time-lapse microscope (Nikon Instruments, Europe). Twenty field positions were recorded covering 4 microwells each. Images were acquired every minute for 2 d to 7 d using a 20X magnitude phase contrast objective. Only microwells containing a single cell were considered in the analyses.

### Image analyses

Images were analysed using ImageJ 1.47g 64-bits software (Rasband, W.S., ImageJ, U.S. National Institutes of Health, Bethesda, Maryland, USA, http://imagej.nih.gov/ij/, 1997–2014). Cell tracking was performed manually using the ImageJ TrackMate plugin. The morphologies of first, second, and third generation cells were analysed semiautomatically with Fiji (ImageJ 1.50e). A cell counter plugin was used to identify the moment when the cell switches from a round to a polarised morphology.

### Analysis of the time-lapse records

Analyses of time-lapse data were performed using R software. Cell lineage representations, cycle length, roundness, and switch frequency were calculated with custom R-made scripts. Euclidean distances of the last 3 parameters (cycle length, roundness, and switch frequency) between the 2 sister cells were calculated. Cells were classified into 2 groups using the k-means algorithm: with similar or divergent dynamic phenotypes. Box plot representation combined with individual points was calculated with the beeswarm package (Aron Eklund [2016]. beeswarm: The Bee Swarm Plot, an Alternative to Stripchart. R package version 0.2.3. https://CRAN.R-project.org/package=beeswarm). The ggplot2 package was used to represent the roundness and switch frequency of cells sorted on CD133 protein.

### Proliferation assay

CD34+ cells were labeled with 2.5 μM of CTV (Life technologies) at t = 0 h and analysed using flow cytometry (LSRII–BD biosciences, France) after 24 h, 48 h, and 72 h with ModFit LT software as described previously by Neildez et al. [57]

### Image flow cytometry

Image flow cytometry analysis was performed using Image Stream MKII (Amnis, Proteigen, Merk Millipore). 5.10^4^ cells were cultured in a 48-well plate in 200 μL prestimulation medium. After 72 h, 100 μL of 3% glutaraldehyde was added to the cell-containing well (1% final) for 15 min. Glutaraldehyde offers good preservation of cell shape. Cells were washed twice with PBS 1X and incubated 2 h with 2 mg/mL NaBH_4_ at room temperature. Fcɤ receptors were saturated with Gamma Immune (Sigma Aldrich) for 5 min at 4°C (1:2 dilution). Cells were labeled for 20 min at 4°C with a 1:10 dilution of mouse anti-human CD133-APC antibody (clone AC133, Miltenyi Biotec). Cells were then suspended in PBS and analysed with the image flow cytometer. Bright Field and APC channels were recorded (Bright Field: 745-nm laser; APC: 642-nm laser) with the 40X magnitude objective. Analyses of image stream data were performed with the IDEAS software (Amnis, Proteigen, Merk Millipore).

### Cell sorting

The CD34^+^CD133^high^, CD34^+^CD133^medium^ and CD34^+^CD133^low/neg^ cells were sorted at t = 48 h. Prior to labeling, Fc receptors were saturated with Gamma Immune (Sigma Aldrich). The CD34+ cells were labeled with CD34-PE (Miltenyi Biotec), CD45-APC-H7 (Beckman Coulter) and CD133-APC (clone AC133, Miltenyi Biotec) antibodies and 7—Aminoactinomycine D (Sigma Aldrich). Isotype controls were used for the gating strategy. Cells were purified using a MoFlo Astrios cell sorter (Beckman Coulter, France) and analysed with Kaluza software.

### Flow cytometric analysis

The CD34+ cells were labeled using the following cell-surface markers: CD34-PE (Miltenyi Biotec), CD38-Pacific Blue (Beckman Coulter), and CD90-APC-Cy7 (Beckman Coulter) antibodies and 7-AAD marker (Sigma Aldrich). Isotype controls were used for gating strategies. Cells were analysed at 72 h after prestimulation by flow cytomety (LSRII–BD biosciences, France) and analysed with FlowJo (v10.1) software.

## Supporting information

S1 Fig Full set of gene expression data obtained using single-cell qRT-PCR in cord-blood CD34+ cells cultured in vivo with early-acting cytokines. Extended heat map of the transcriptional profiles of cord blood-derived CD34+ cells at t = 0h, t = 24h, t = 48h and t = 72h after the beginning of the experiment. The color codes for the time points of cells are indicated on the right, the color code for expression level are indicated below the heat-map. Note the tendency of cells with the same time-points to cluster. (Underlying data can be found in S1 Data.).
(TIF)

S2 Fig Violin plot representation of individual gene expression levels at the four time points. (Underlying data can be found in S1 Data.).
(TIF)

S3 Fig Principal component analysis of single-cell expression profiles. A. 2D PCA plot. Each point represents a single cell and the different time-points are coloured differently. Color codes are in the box to the right of the plot. B. Contribution of individual genes to principal component 1 and 2. Only the 40 highest contributions are indicated. (Underlying data can be found in S1 Data.).
(TIF)

S4 Fig Analysis of cell division rates. A. The number of cells at t = 24h, t = 48h and t = 72h as observed by time-lapse microscopy. The cells of different generations are color coded in the histogram. Note that none of the cells has divided after 24 hours and only 11 of the 32 cells underwent one division after 48 hours. At t = 72h, three of the founder cells have not undergone division. (Underlying data can be found in S2 Data) B. Cell division analysis using Cell Trace Violet labelling. Cells were labelled at t = 0h (not shown) and analyzed using flow cytometry at t = 24h, t = 48h and t = 72h. When divided, the average fluorescence intensity of the two daughter cells is reduced by half compared to the maternal cell. Therefore, the peak on the right represents the parental generation. The number of the peaks to the left indicates the number of cell generations in the culture and the size of the peaks is indicative of the number of cells in each generation. Note that after 24h no cell division is detected and after 72h a fraction of undivided cells can still be detected. Most of the cells underwent one or two divisions. Overall, the profile is very similar to that detected by time lapse. (Underlying data can be found in S3 Data.).
(TIF)

S5 Fig Representations of morphological profiles of cells in three representative clones. Each horizontal box in the three panels represents the morphology of an individual cell. The cell morphology–polarized or round–is shown with a horizontal line, the length of which is proportional to the time spent in the corresponding form. Vertical lines show the transitions between forms. The length of the horizontal lines is proportional to duration of the cell cycle and the time scale in hours is the same for each cell. The founder cell is numbered Cell_1, the two daughter cells Cell_11 and Cell_12 and granddaughter cell pairs as Cell_111, Cell_112 and Cell_121, Cell_122 respectively. In clone number 1 the polarized founder cell gives rise to frequent switcher daughters and granddaughters. Note the striking similarity of the time profiles for the morphological switches that can be observed in sister cells. In clone number 2 the polarized founder cell gives rise to stable polarized siblings. In clone number 3 the founder cell and its progeny are round. The two daughter cells switch to polarized shape for short periods. Note again the striking similarity of the sister cells’ switch profiles. (Underlying data can be found in S2 Data.).
(TIF)

S6 Fig Cell morphology and CD133 localisation. Image-based cytometry analysis shows correlation between the CD133 protein expression level and cell morphology at t = 72h. The middle plot shows the CD133 protein density detected in glutaraldehyde-fixed cells. Representative examples of the morphologies of “high” (upper frame) and “low” (lower frame) expressing cells are shown on the left and right respectively.
(TIF)

S7 Fig The full set of the gene expression data obtained on “high,” “medium,” and “low” CD133 expressing individual cells. A. Heat-map representation of the expression levels of 90 genes as determined by single-cell qRT-PCR. Color codes for the “high”, “medium” and “low” fractions are indicated on the right, and the color codes for expression levels are indicated below the heat-map. Note the intermediate expression pattern of the “medium” cells. B. Principal component analysis of the single-cell gene expression data shown on the panel A. “Medium” cells are intermediate. (Underlying data can be found in S1 Data.).
(TIF)

S8 Fig Violin plot representation of individual gene expression levels in the “high,” “medium,” and “low” CD133 cells. The color code is identical to that on S7 Fig. (Underlying data can be found in S1 Data.).
(TIF)

S9 Fig Cytometry analysis of the effects of valproic acid on CD34+ cells. The histogram in the left panel indicates the proportion of CD34+/CD38- cells in VPA+ and VPA- cell cultures at different time points. Note that there is no substantial difference between the two. The right panel indicates the proportion of CD34+/CD90+ cells in the same cultures. Note the increasing proportion of CD34+/CD90+ cells in VPA+ culture. This rapid increase cannot be explained by the selective proliferation of the CD90+ cells and is the result of the de novo synthesis of the CD90 protein, because as indicated in Fig 2, and S4 Fig, cells do not divide before 72h. (Underlying data can be found in S3 Data.).
(TIF)

S1 Table List of genes analyzed and primer sequences used for single-cell qRT-PCR amplification. (XLSX)

S1 Data RTqPCR normalized. (XLSX)

S2 Data Timelapse. (XLSX)

S3 Data Cytometry. (XLSX)

S1 Movie Time-lapse video of a cell clone with cells conserving polarized morphologies. The video has been accelerated to 5 frames per second.
(MOV)

S2 Movie Time-lapse video of a cell clone with cells conserving round morphologies. The video has been accelerated to 5 frames per second.
(MOV)

S3 Movie Time-lapse video of a cell clone with cells changing morphology at high frequency (dynamic phenotype of frequent switchers). Only a period between 61 and 81 h is shown. Note that individual snapshots taken at different moments may show a population composed of only polarized, only round or cells with mixed morphology. The video is the original speed.
(MOV)

## Abbreviations

CLP: common lymphoid progenitor
CMP: common myeloid progenitor
CTV: CellTrace Violet
HDAC: histone deacetylase
HSPC: hematopoietic stem and progenitor cell
MPP: multipotent progenitor
PCA: principal component analysis
qRT-PCR: quantitative reverse transcription polymerase chain reaction
t-SNE: t-distributed stochastic neighbour embedding
VPA: valproic acid
