# Supplementary figures and images for: Integrated time-lapse and single-cell transcription studies highlight the variable and dynamic nature of human hematopoietic cell fate commitment
Source: PLoS Biol. 2017 Jul 27;15(7):e2001867. doi: 10.1371/journal.pbio.2001867 (PMC5531424; doi:10.1371/journal.pbio.2001867)

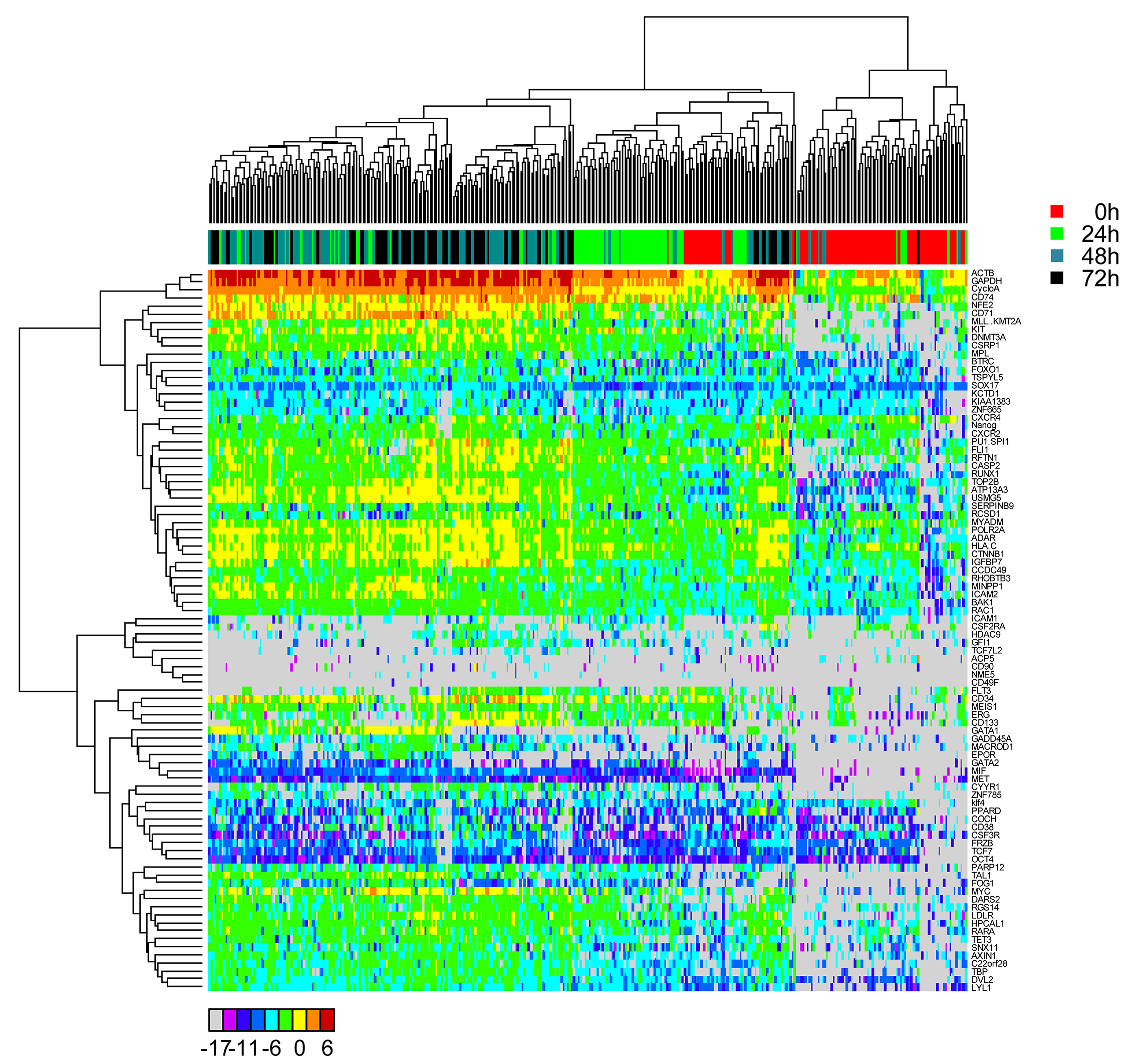

Supplement: S1 Fig — Extended heat map of the transcriptional profiles of cord blood-derived CD34+ cells at t = 0h, t = 24h, t = 48h and t = 72h after the beginning of the experiment. The color codes for the time points of cells are indicated on the right, the color code for expression level are indicated below the heat-map. Note the tendency of cells with the same time-points to cluster. (Underlying data can be found in S1 Data.). (TIF) [file pbio.2001867.s001.tif]

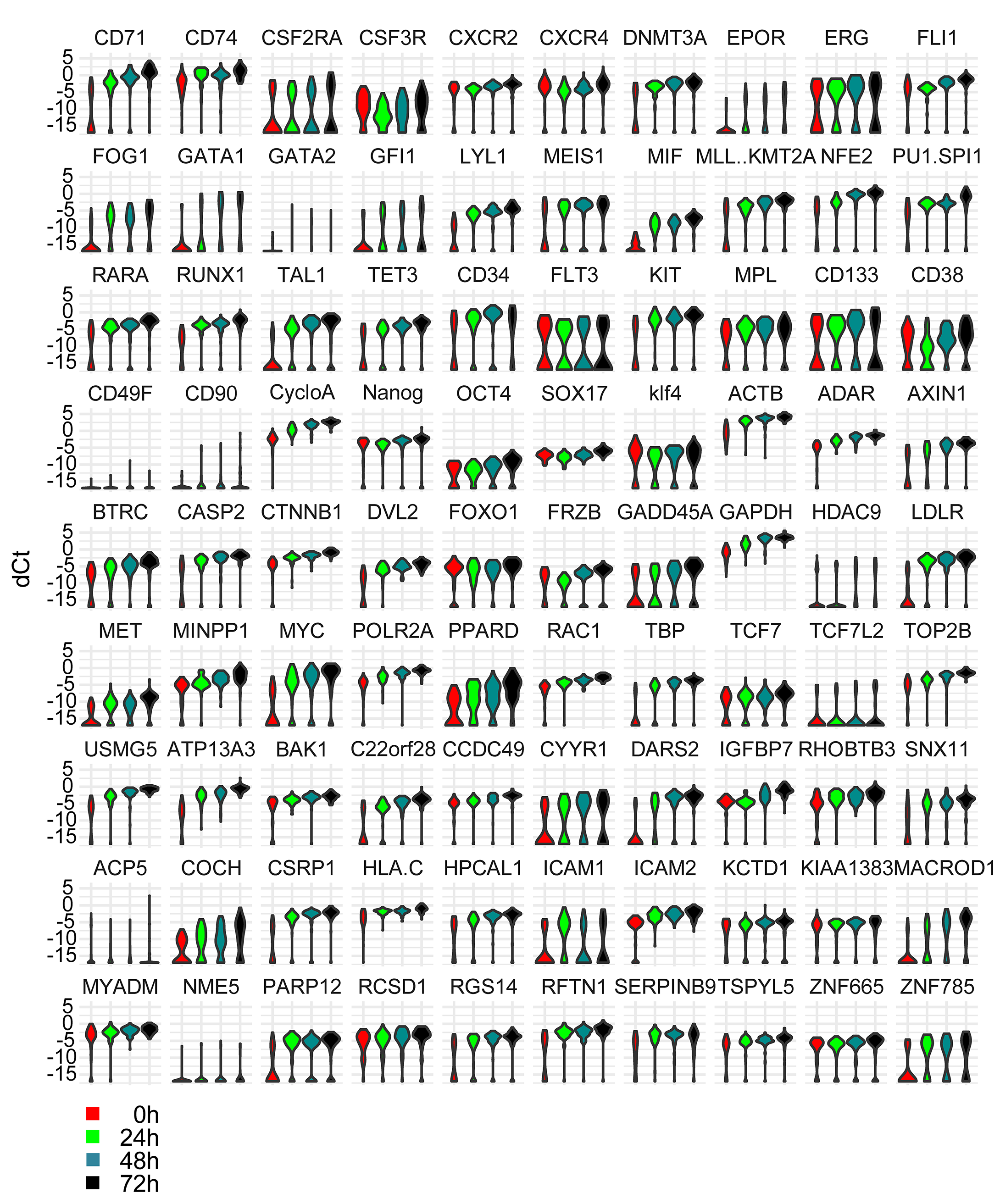

Supplement: S2 Fig — (Underlying data can be found in S1 Data.). (TIF) [file pbio.2001867.s002.tif]

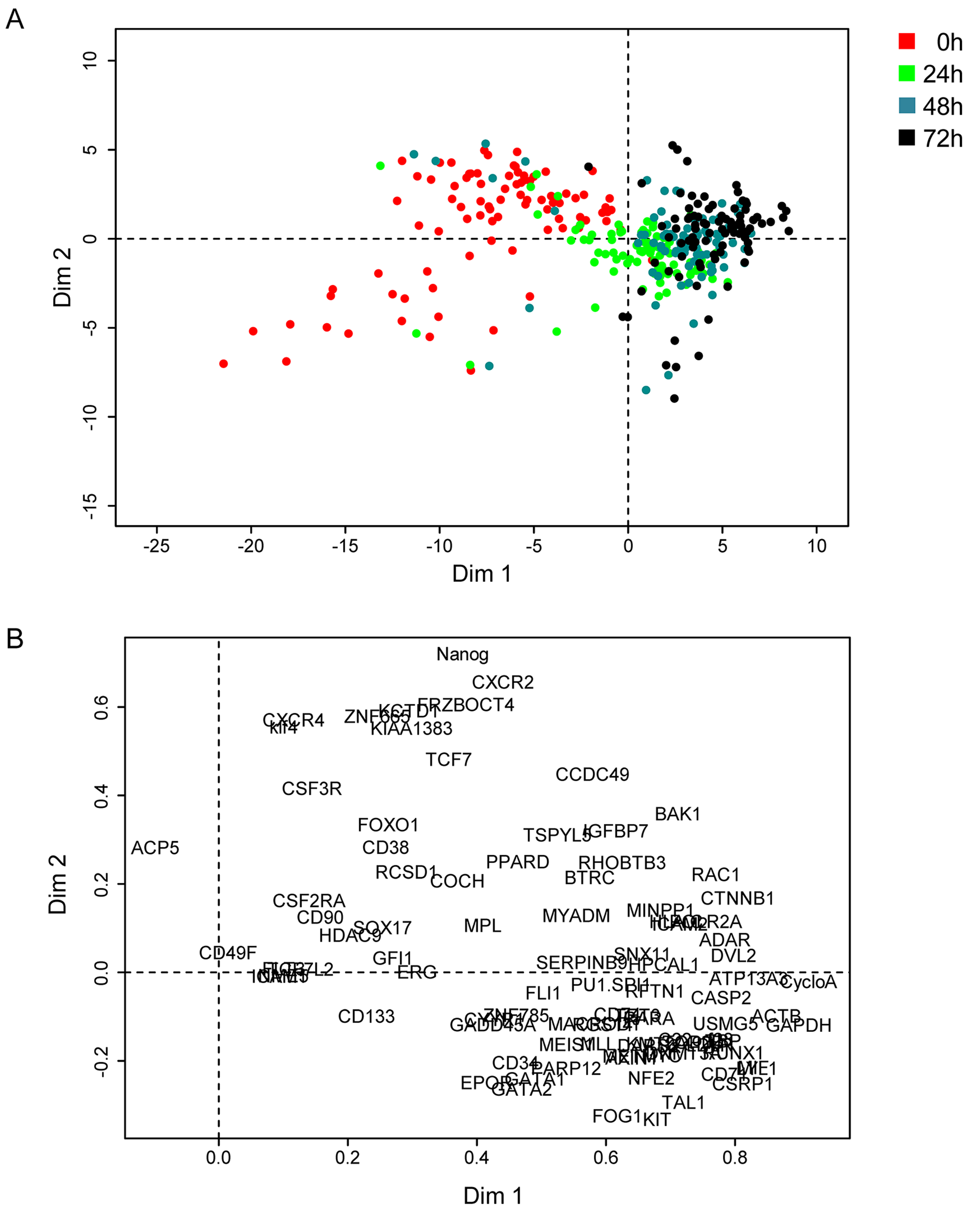

Supplement: S3 Fig — A. 2D PCA plot. Each point represents a single cell and the different time-points are coloured differently. Color codes are in the box to the right of the plot. B. Contribution of individual genes to principal component 1 and 2. Only the 40 highest contributions are indicated. (Underlying data can be found in S1 Data.). (TIF) [file pbio.2001867.s003.tif]

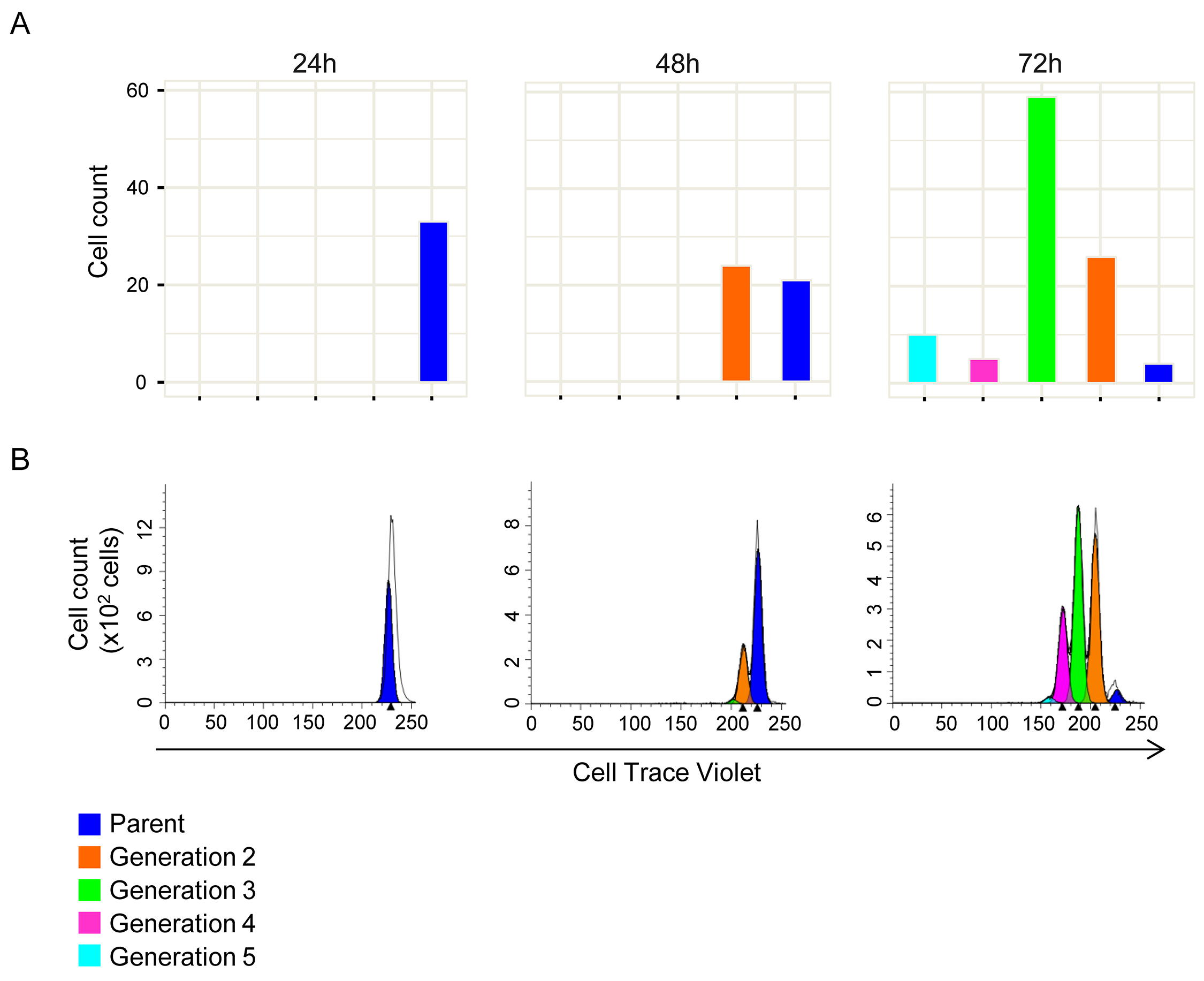

Supplement: S4 Fig — A. The number of cells at t = 24h, t = 48h and t = 72h as observed by time-lapse microscopy. The cells of different generations are color coded in the histogram. Note that none of the cells has divided after 24 hours and only 11 of the 32 cells underwent one division after 48 hours. At t = 72h, three of the founder cells have not undergone division. (Underlying data can be found in S2 Data) B. Cell division analysis using Cell Trace Violet labelling. Cells were labelled at t = 0h (not shown) and analyzed using flow cytometry at t = 24h, t = 48h and t = 72h. When divided, the average fluorescence intensity of the two daughter cells is reduced by half compared to the maternal cell. Therefore, the peak on the right represents the parental generation. The number of the peaks to the left indicates the number of cell generations in the culture and the size of the peaks is indicative of the number of cells in each generation. Note that after 24h no cell division is detected and after 72h a fraction of undivided cells can still be detected. Most of the cells underwent one or two divisions. Overall, the profile is very similar to that detected by time lapse. (Underlying data can be found in S3 Data.). (TIF) [file pbio.2001867.s004.tif]

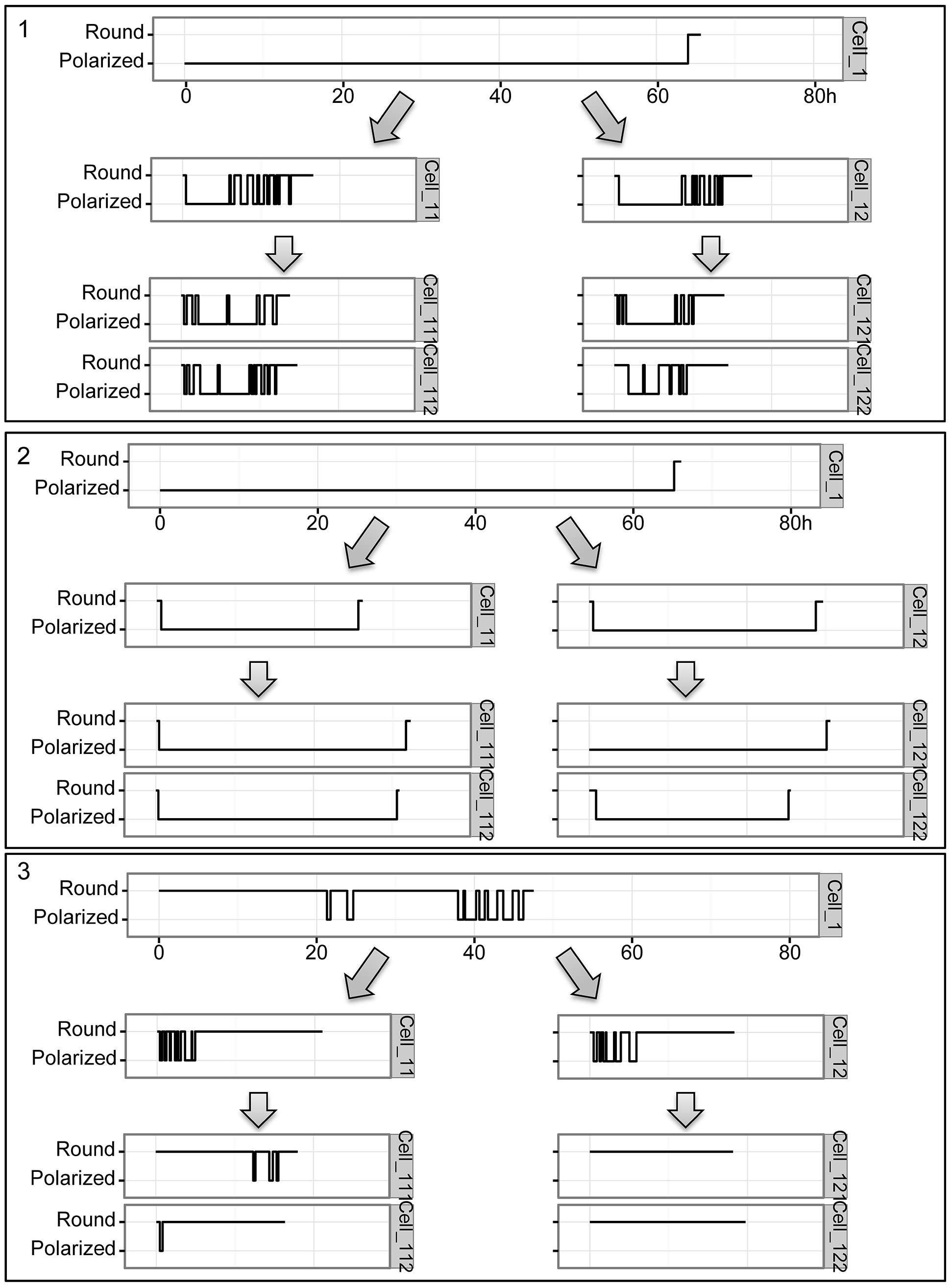

Supplement: S5 Fig — Each horizontal box in the three panels represents the morphology of an individual cell. The cell morphology–polarized or round–is shown with a horizontal line, the length of which is proportional to the time spent in the corresponding form. Vertical lines show the transitions between forms. The length of the horizontal lines is proportional to duration of the cell cycle and the time scale in hours is the same for each cell. The founder cell is numbered Cell_1, the two daughter cells Cell_11 and Cell_12 and granddaughter cell pairs as Cell_111, Cell_112 and Cell_121, Cell_122 respectively. In clone number 1 the polarized founder cell gives rise to frequent switcher daughters and granddaughters. Note the striking similarity of the time profiles for the morphological switches that can be observed in sister cells. In clone number 2 the polarized founder cell gives rise to stable polarized siblings. In clone number 3 the founder cell and its progeny are round. The two daughter cells switch to polarized shape for short periods. Note again the striking similarity of the sister cells’ switch profiles. (Underlying data can be found in S2 Data.). (TIF) [file pbio.2001867.s005.tif]

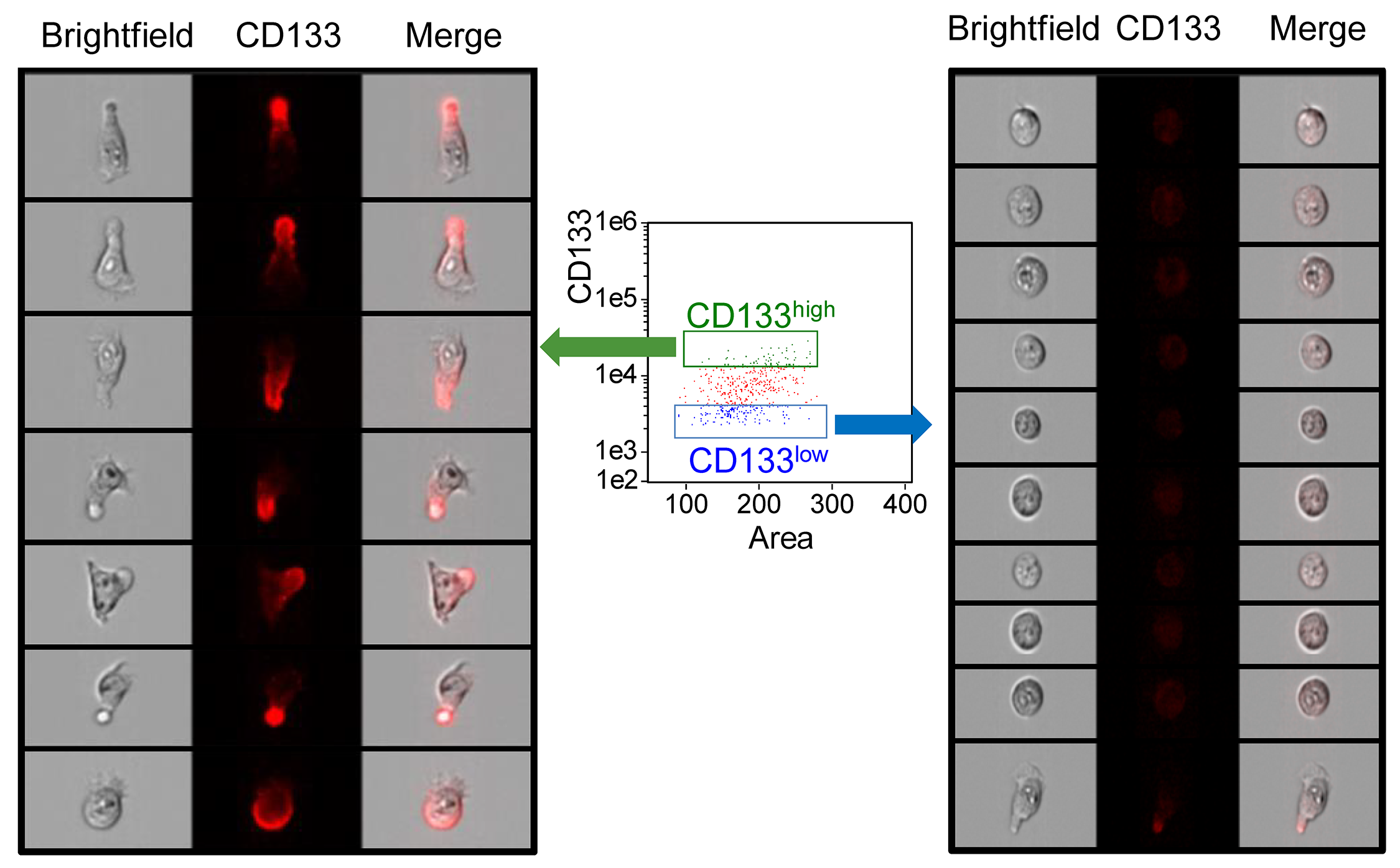

Supplement: S6 Fig — Image-based cytometry analysis shows correlation between the CD133 protein expression level and cell morphology at t = 72h. The middle plot shows the CD133 protein density detected in glutaraldehyde-fixed cells. Representative examples of the morphologies of “high” (upper frame) and “low” (lower frame) expressing cells are shown on the left and right respectively. (TIF) [file pbio.2001867.s006.tif]

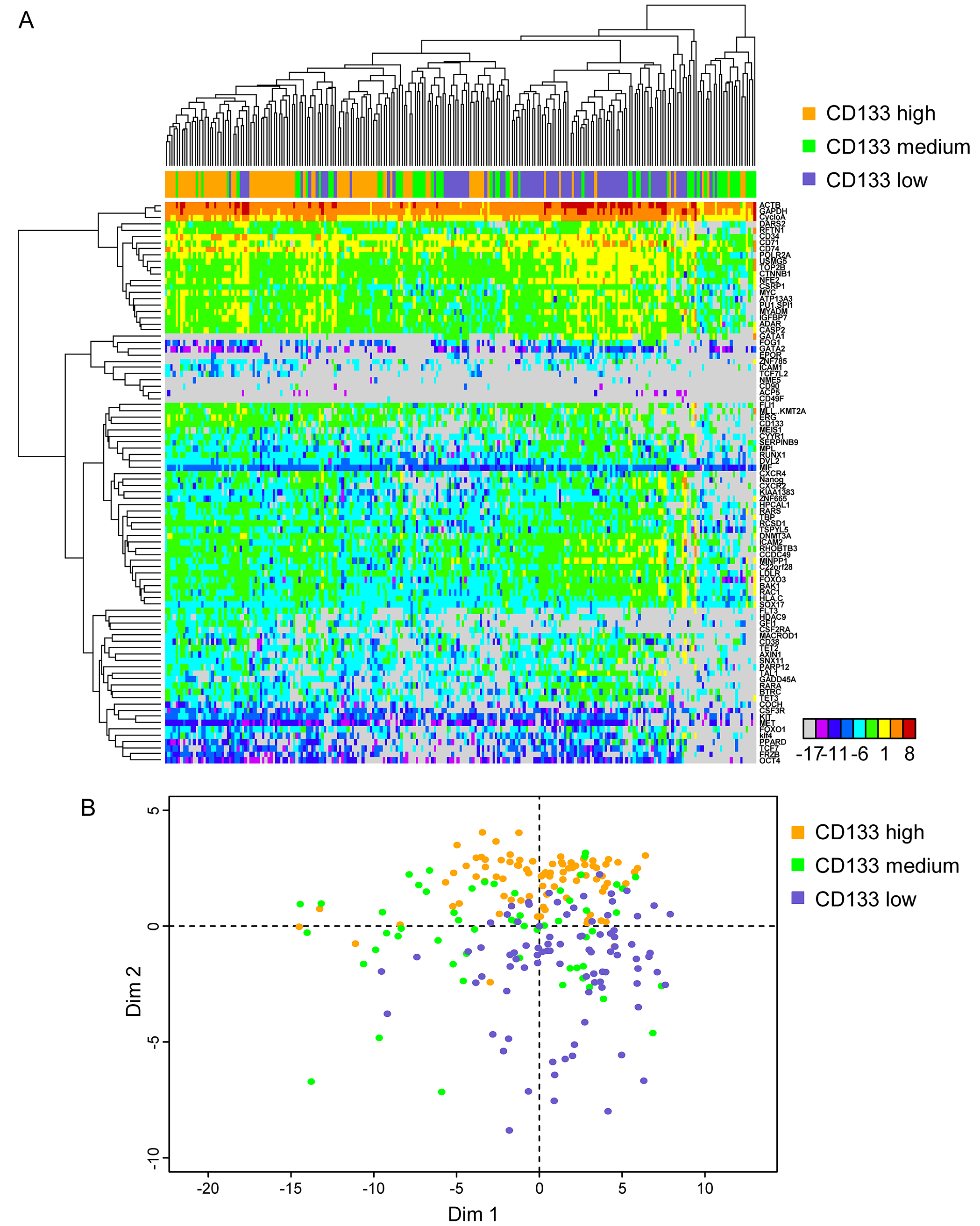

Supplement: S7 Fig — A. Heat-map representation of the expression levels of 90 genes as determined by single-cell qRT-PCR. Color codes for the “high”, “medium” and “low” fractions are indicated on the right, and the color codes for expression levels are indicated below the heat-map. Note the intermediate expression pattern of the “medium” cells. B. Principal component analysis of the single-cell gene expression data shown on the panel A. “Medium” cells are intermediate. (Underlying data can be found in S1 Data.). (TIF) [file pbio.2001867.s007.tif]

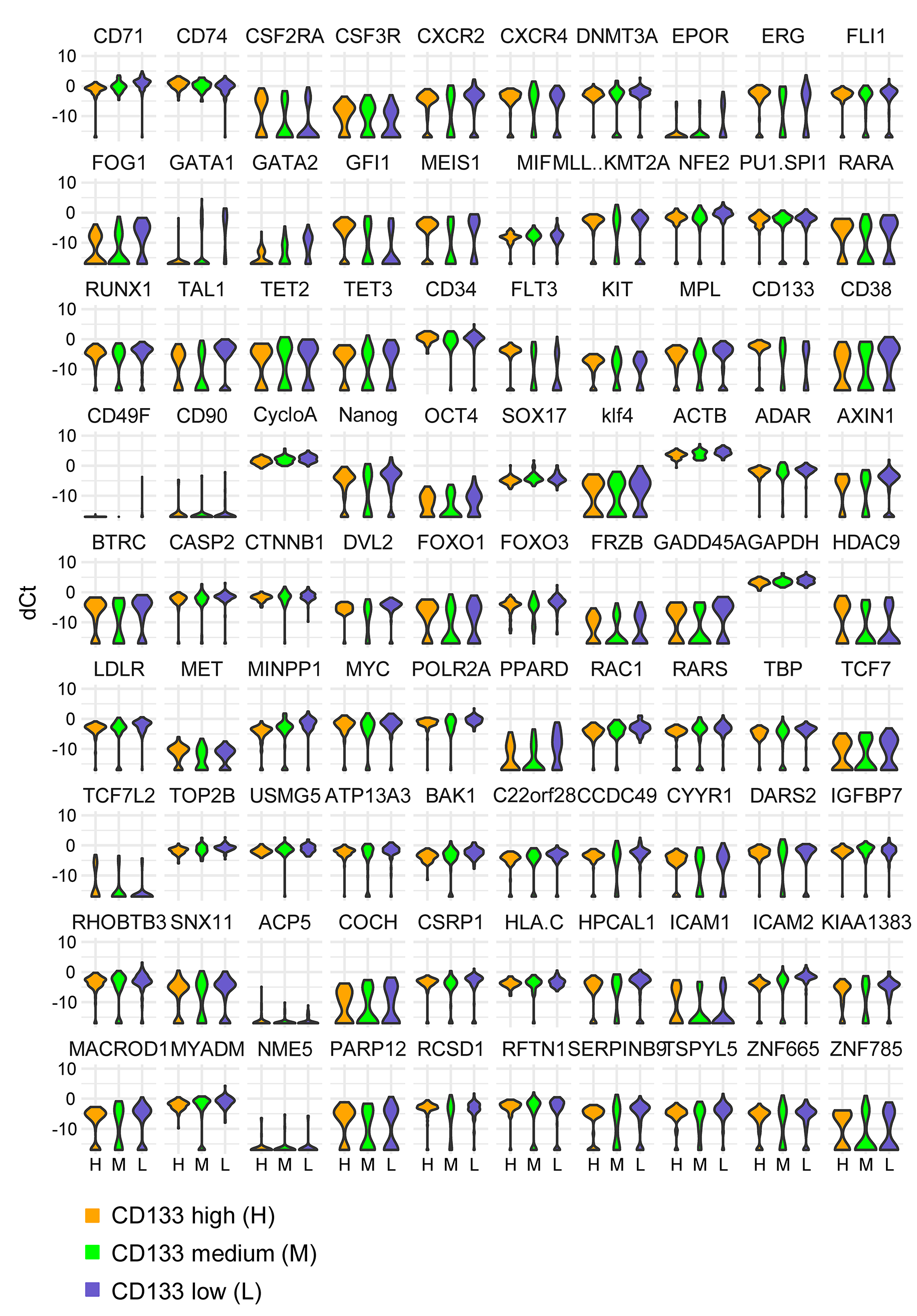

Supplement: S8 Fig — The color code is identical to that on S7 Fig. (Underlying data can be found in S1 Data.). (TIF) [file pbio.2001867.s008.tif]

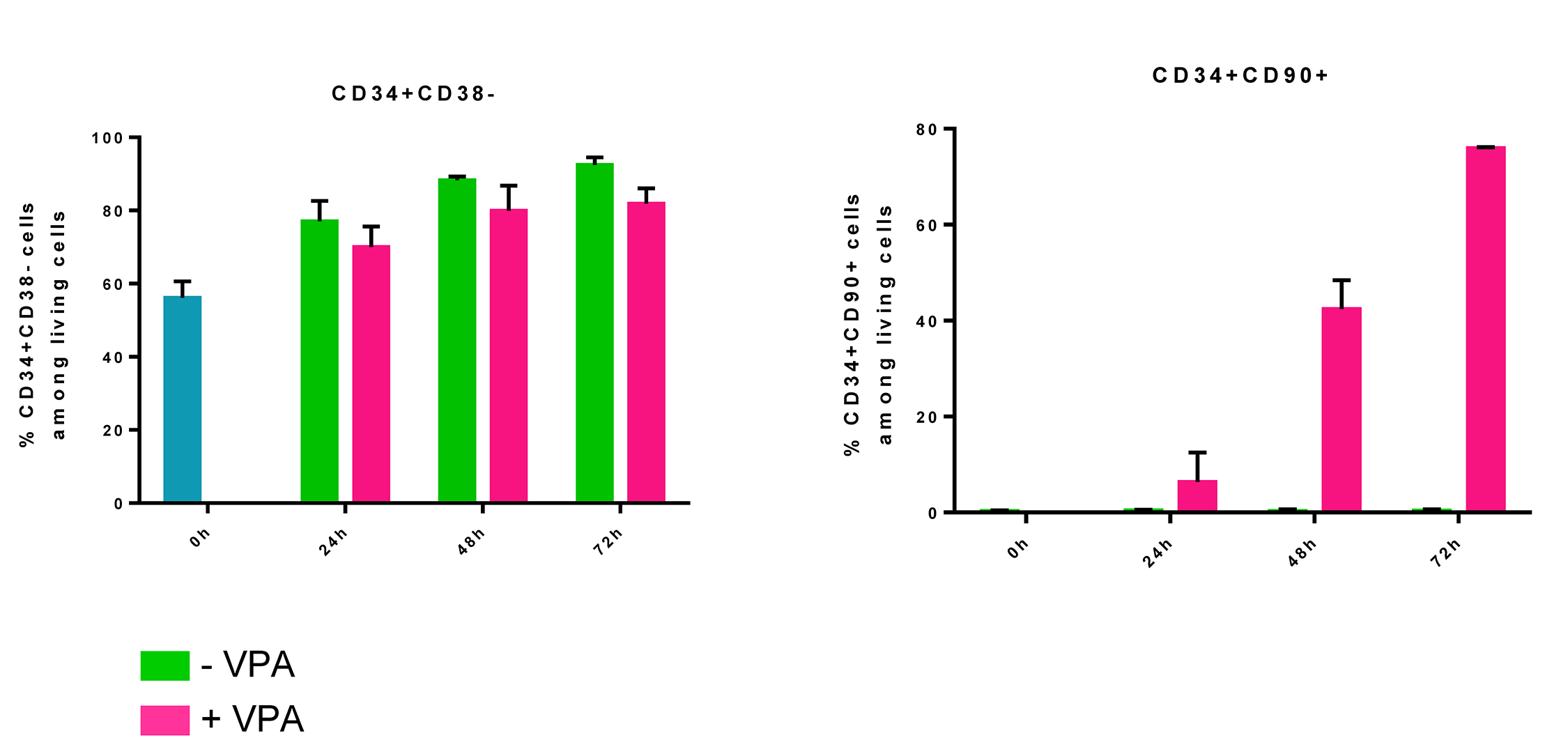

Supplement: S9 Fig — The histogram in the left panel indicates the proportion of CD34+/CD38- cells in VPA+ and VPA- cell cultures at different time points. Note that there is no substantial difference between the two. The right panel indicates the proportion of CD34+/CD90+ cells in the same cultures. Note the increasing proportion of CD34+/CD90+ cells in VPA+ culture. This rapid increase cannot be explained by the selective proliferation of the CD90+ cells and is the result of the de novo synthesis of the CD90 protein, because as indicated in Fig 2, and S4 Fig, cells do not divide before 72h. (Underlying data can be found in S3 Data.). (TIF) [file pbio.2001867.s009.tif]
